# The global survival rate among adult out-of-hospital cardiac arrest patients who received cardiopulmonary resuscitation: a systematic review and meta-analysis

**DOI:** 10.1186/s13054-020-2773-2

**Published:** 2020-02-22

**Authors:** Shijiao Yan, Yong Gan, Nan Jiang, Rixing Wang, Yunqiang Chen, Zhiqian Luo, Qiao Zong, Song Chen, Chuanzhu Lv

**Affiliations:** 1 School of Public Health, Hainan Medical University, Haikou, Hainan China; 2 Key Laboratory of Emergency and Trauma of Ministry of Education, Hainan Medical University, Haikou, Hainan China; 3 Department of Social Medicine and Health Management, School of Public Health, Tongji Medical College, Huazhong University of Science and Technology, Wuhan, Hubei China; 4 Department of Emergency, Hainan Clinical Research Center for Acute and Critical Diseases, The Second Affiliated Hospital of Hainan Medical University, Haikou, Hainan China; 5 Emergency and Trauma College, Hainan Medical University, Haikou, Hainan China; 6 School of International Education, Hainan Medical University, Haikou, Hainan China; 7 Department of Emergency, the First Affiliated Hospital of Hainan Medical University, No.3 Xueyuan Road, Longhua Zone, Haikou, 571199 China

**Keywords:** Out-of-hospital cardiac arrest, Resuscitation, Emergency medical services

## Abstract

**Background:**

To quantitatively summarize the available epidemiological evidence on the survival rate of out-of-hospital cardiac arrest (OHCA) patients who received cardiopulmonary resuscitation (CPR).

**Methods:**

We systematically searched the PubMed, Embase, and Web of Science databases, and the references of retrieved articles were manually reviewed to identify studies reporting the outcome of OHCA patients who received CPR. The overall incidence and outcome of OHCA were assessed using a random-effects meta-analysis.

**Results:**

A total of 141 eligible studies were included in this meta-analysis. The pooled incidence of return of spontaneous circulation (ROSC) was 29.7% (95% CI 27.6–31.7%), the rate of survival to hospital admission was 22.0% (95% CI 20.7–23.4%), the rate of survival to hospital discharge was 8.8% (95% CI 8.2–9.4%), the pooled 1-month survival rate was 10.7% (95% CI 9.1–13.3%), and the 1-year survival rate was 7.7% (95% CI 5.8–9.5%). Subgroup analysis showed that survival to hospital discharge was more likely among OHCA patients whose cardiac arrest was witnessed by a bystander or emergency medical services (EMS) (10.5%; 95% CI 9.2–11.7%), who received bystander CPR (11.3%, 95% CI 9.3–13.2%), and who were living in Europe and North America (Europe 11.7%; 95% CI 10.5–13.0%; North America: 7.7%; 95% CI 6.9–8.6%). The survival to discharge (8.6% in 1976–1999 vs. 9.9% in 2010–2019), 1-month survival (8.0% in 2000–2009 vs. 13.3% in 2010–2019), and 1-year survival (8.0% in 2000–2009 vs. 13.3% in 2010–2019) rates of OHCA patients who underwent CPR significantly increased throughout the study period. The Egger’s test did not indicate evidence of publication bias for the outcomes of OHCA patients who underwent CPR.

**Conclusions:**

The global survival rate of OHCA patients who received CPR has increased in the past 40 years. A higher survival rate post-OHCA is more likely among patients who receive bystander CPR and who live in Western countries.

**Electronic supplementary material:**

The online version of this article (10.1186/s13054-020-2773-2) contains supplementary material, which is available to authorized users.

## Background

Out-of-hospital cardiac arrest (OHCA) is a major public health challenge, with an average global incidence among adults of 55 OHCAs per 100,000 person-years. [1] In China, there are more than 230 million people with cardiovascular disease, and 550,000 individuals experience cardiac arrest every year [2]. Worldwide, survival after OHCA remains poor [3]. In China, the survival rate of OHCA is less than 1% [4]. Early initiation, good cardiopulmonary resuscitation (CPR) quality, and the use of an automated external defibrillator (AED) significantly improved survival and long-term outcomes in survivors of OHCA [2, 5–7].

Many studies have been conducted to estimate the survival rate among OHCA patients who received CPR in different regions of the world [4, 8–13]. However, the results were inconsistent across studies. The purpose of this systematic review and meta-analysis was to estimate the overall incidence of the return of spontaneous circulation (ROSC), the survival to admission rate, the survival to discharge rate, the 1-month survival rate, and the 1-year survival rate of patients after OHCA who received CPR worldwide.

## Methods

This systematic review and meta-analysis adhered to the Preferred Reporting Items for Systematic Reviews and Meta-Analyses (PRISMA) guidelines [14] and the Meta-analysis of Observational Studies in Epidemiology (MOOSE) checklist [15].

### Search strategy

Studies that reported survival rates among OHCA patients who underwent CPR were identified from the PubMed, Embase, and Web of Science databases from their inception to February 2019. The search terms were “out-of-hospital cardiac arrest” or “out-of-hospital ventricular fibrillation/ventricular tachycardia/asystole/pulseless electrical activity” or “cardiopulmonary resuscitation” or “CPR” or “mouth to mouth” or “resuscitation” or “resuscitation orders” or “survival” or “survival rate” or “mortality” or “sudden cardiac death”. Only articles published in English were considered. Additionally, we manually reviewed the references listed in the retrieved articles to identify additional pertinent publications.

### Inclusion criteria and exclusion criteria

Studies were included if they met the following eligibility criteria: (1) the study design was based on the Utstein-style reporting guidelines; (2) the study population was composed of adults, which included any study in which less than 20% of study population were pediatric patients (age < 18 years); (3) the outcome variables were at least one of the following: ROSC, survival to admission rate, survival to hospital discharge rate, 1-month survival rate, and 1-year survival rate; (4) cardiac arrest happened outside the hospital; and (5) the study design was prospective, retrospective, or interventional. Reviews, letters, editorials, guidelines, and case reports were excluded. When multiple publications were produced using the same study population, the most recent and informative paper was included.

### Data extraction

Two independent reviewers (YG and NJ) performed the data extraction. The following data were extracted from the studies: the first author’s name, region of population, year of publication, sex, number of cardiac arrests and survivors, cardiac arrest witness type, provision of CPR, and origin of cardiac arrest. The rate of survival to hospital discharge was considered the primary outcome; ROSC, the rate of survival to hospital admission, 1-month survival rate and 1-year survival rate were also analyzed as outcome variables. Any disagreements between the investigators were discussed, and an agreement was reached through consensus.

### Statistical analysis

A random-effects model was used to estimate the survival rates among OHCA patients who received CPR [16]. Studies that reported the survival outcomes of OHCA patients who received CPR (ROSC, survival to admission rate, survival to discharge rate, 1-month survival rate, and 1-year survival rate) were treated as independent reports.

Statistical heterogeneity across studies was assessed with the *I*^*2*^ statistic, where values of 25%, 50%, and 75% represented the cut-off points for low, moderate, and high levels of heterogeneity, respectively [17]. Publication bias was evaluated with funnel plots and Egger’s test [18]. Subgroup analyses stratified by sex, study location, study period, origin of OHCA, CPR type, and cardiac arrest witness type were conducted to investigate potential sources of heterogeneity across subgroups and examine the robustness of the primary results. We performed sensitivity analyses by omitting one study at a time to assess the influence of any single study on the pooled survival rate estimates. All statistical analyses were conducted with STATA V.12.0 (StataCorp, College Station, TX). All tests were two-tailed with a significance level of 0.05.

## Results

### Study selection

**Fig. 1:**
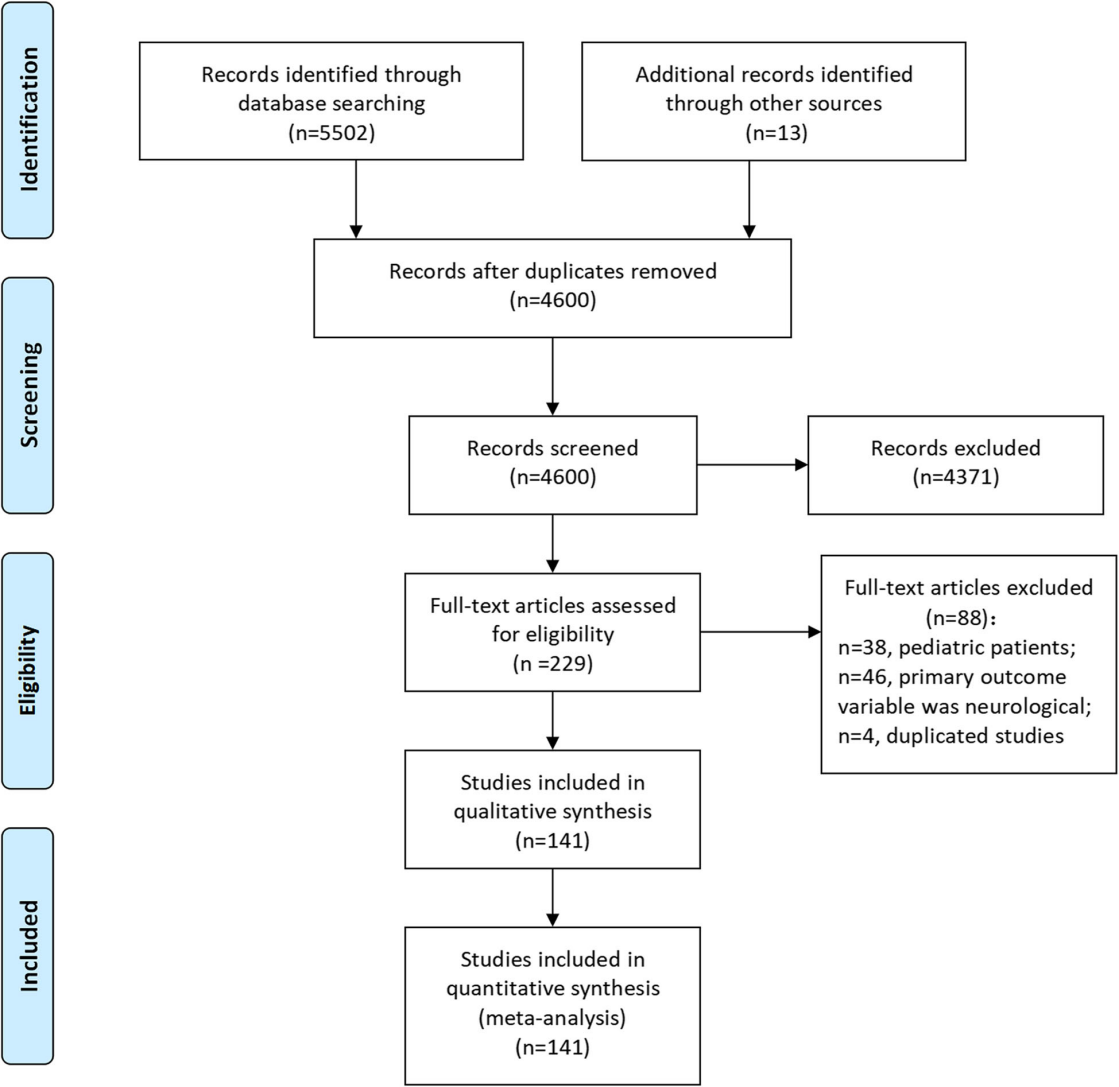
Flow chart of relevant study identification in relation to outcomes of OHCA who underwent CPR

The process of study selection, identification, and inclusion using the Preferred Reporting Items for Systematic Reviews and Meta-Analyses flow diagram is presented in Fig. 1. Initially, 5502 articles were retrieved from the PubMed, Embase, and Web of Science databases. In addition, we identified 13 articles by manually searching the reference lists of the retrieved articles. After removing 915 duplicate articles, we further excluded 4371 articles based on the titles and abstracts, including 336 nonhuman studies and 280 reviews, editorials, letters, guidelines or case reports. A total of 229 articles were selected for further full texts assessment. After retrieving the full-text for evaluation in detail, 88 articles were excluded because their study populations were pediatric patients, their primary outcome focused on the neurological survival rate or they were multiple publications produced using the same study population. Finally, a total of 141 studies were included in the present meta-analysis. The references of the studies included in the meta-analysis are listed in the Additional file 1.

### Characteristics and quality of the studies included

The characteristics of the 141 included studies are shown in Supplementary Table 1. These studies were conducted on 4 continents. Fifty-six studies were conducted in Europe, 48 in North America, 29 in Asia, 6 in Oceania, and two in both Europe and North America. A total of 31 studies reported the survivors and study population stratified by sex. The year of publication ranged from 1976 to 2019. In total, 4,610,669 OHCA patients who underwent CPR were involved in this study. We included 62 studies that reported the incidence of ROSC, 88 studies that explored the rate of survival to admission, 103 studies that assessed the rate of survival to discharge, 33 studies that investigated the 1-month survival rate, and 22 studies that reported the 1-year survival rate. Nineteen studies included pediatric patients, but less than 20% of study population was younger than 18 years.

### ROSC

In this study, 62 studies with 74 reports reported the outcome of ROSC in OHCA patients who underwent CPR. From the random-effects meta-analysis, we found that the pooled incidence of ROSC among OHCA patients was 29.7% (95% CI, 27.6–31.7%) with significant heterogeneity across studies (*I*^2^ = 99.9%, *P* < 0.001).

Subgroup analyses showed significant differences in the incidence of prehospital ROSC by study location, the provider of CPR, and study period (Table 1). With regard to study location, Oceania had the highest incidence of ROSC (38.6%; 95% CI 22.9–54.2%), followed by Europe (36.7%; 95% CI 32.4–40.9%). Asia had the lowest incidence of ROSC (22.1%; 95% CI 18.1–26.0%). Regarding the provider of CPR, the incidence of ROSC was the highest among OHCA patients who had received CPR from emergency medical services (EMS) (36.3%; 95% CI 23.8–48.9%).

**Table 1:** The incidence of ROSC, survival to admission, survival to discharge, 1 month survival, and 1 year survival: the overall estimates and subgroup analyses

	No. of reports	Survivors	OHCA cases	Proportion (%)	95%CI	*I*^2^	*P* value for heterogeneity
ROSC*	**74**	**203,084**	**1,560,830**	**29.7**	**27.6 to 31.7**	**99.90%**	**< 0.001**
**Sex**							
Men	2	242	856	28.9	24.2 to 33.6	43.4%	0.184
Women	2	151	478	31.6	27.4 to 35.8	0.00%	0.542
Combined	70	202,691	1,559,496	29.7	27.6 to 31.8	99.90%	< 0.001
**Study location**							
Europe	32	4166	12,274	36.7	32.4 to 40.9	96.10%	< 0.001
Asia	17	186,060	1,500,110	22.1	18.1 to 26.0	100.00%	< 0.001
North America	20	6981	32,520	24.3	19.7 to 28.9	99.10%	< 0.001
Oceania	5	5877	15,926	38.6	22.9 to 54.2	99.20%	< 0.001
**Study period**							
1976–1999	25	3706	15,429	34.2	28.0 to 40.4	98.70%	< 0.001
2000–2009	30	4091	17,159	28.3	23.9 to 32.7	98.00%	< 0.001
2010–2019	19	195,287	1,528,242	27.5	23.6 to 31.3	100.00%	< 0.001
**CPR type**							
Bystander CPR	17	138,149	863,978	26.3	20.9 to 31.8	100.00%	< 0.001
EMS CPR	15	45,505	624,496	36.3	23.8 to 48.9	99.50%	< 0.001
Unspecific CPR	42	19,430	72,356	29.6	25.9 to 33.3	99.30%	< 0.001
**Origin of OHCA**							
Cardiac etiology	49	30,531	143,831	33.9	30.1 to 37.7	99.50%	< 0.001
Non-traumatic	14	7652	33,318	21.5	16.2 to 26.8	99.20%	< 0.001
All patients	11	164,901	1,383,681	23.3	18.3 to 28.3	100.00%	< 0.001
Others	NA	NA	NA	NA	NA	NA	NA
**Witnessed type**							
Witnessed	31	106,894	440,281	36.4	30.6 to 42.2	99.90%	< 0.001
Not witnessed	1	34	93	36.6	26.8 to 46.4	0.00%	< 0.001
Mixed	36	95,828	1,119,248	23.7	22.2 to 25.3	99.80%	< 0.001
Unspecific events	6	328	1208	28.2	18.6 to 37.7	91.30%	< 0.001
**Survival to admission**†	**122**	**55,026**	**377,727**	**22.0**	**20.7 to 23.4**	**99.4%**	**< 0.001**
**Sex**							
Men	7	564	2829	20.3	16.3 to 24.3	81.00%	< 0.001
Women	7	254	1021	23.4	17.8 to 29.1	72.60%	< 0.001
Combined	108	54,208	373,877	22.1	20.6 to 23.5	99.40%	< 0.001
**Study location**							
Europe	52	20,987	123,024	25.7	23.9 to 27.6	98.30%	< 0.001
Asia	27	23,551	203,283	15.6	13.2 to 18.0	99.70%	< 0.001
North America	39	5504	35,183	20.5	18.1 to 22.9	97.50%	< 0.001
Oceania	4	4984	16,237	33.5	21.7 to 45.3	98.90%	< 0.001
**Study period**							
1976–1999	59	5704	33,083	22.4	20.0 to 24.8	97.30%	< 0.001
2000–2009	38	18,433	106,336	25.1	22.9 to 27.3	98.50%	< 0.001
2010–2019	25	30,889	238,308	17.2	14.4 to 19.9	99.80%	< 0.001
**CPR type**							
Bystander CPR	21	6660	44,028	22.8	18.8 to 26.8	99.10%	< 0.001
EMS CPR	23	2043	7657	25.5	19.7 to 31.2	97.40%	< 0.001
Unspecific CPR	78	46,323	326,042	21.1	19.5 to 22.7	99.50%	< 0.001
**Origin of OHCA**							
Cardiac etiology	66	22,682	143,612	23.5	21.2 to 25.9	99.50%	< 0.001
Non-traumatic	38	17,293	142,532	19.3	17.5 to 21.2	98.60%	< 0.001
All patients	11	13,953	86,443	20.4	17.1 to 23.7	99.10%	< 0.001
Others	7	1098	5140	23.6	19.4 to 27.7	91.50%	< 0.001
**Witnessed type**							
Witnessed	37	12,027	88,992	23.2	20.3 to 26.1	99.60%	< 0.001
Not witnessed	1	23	93	24.7	15.9 to 33.5	0.00%	< 0.001
Mixed	75	42,630	286,470	21.6	20.1 to 23.1	99.10%	< 0.001
Unspecific events	9	346	2172	19.2	14.5 to 23.9	84.30%	< 0.001
**Survival to discharge**^**‡**^	**168**	**20,946**	**267,862**	**8.8**	**8.2 to 9.4**	**97.60%**	**< 0.001**
**Sex**							
Men	22	1576	26,666	7.4	6.2 to 8.7	93.60%	< 0.001
Women	21	723	13,570	7.2	5.6 to 8.7	91.70%	< 0.001
Combined	125	18,647	227,626	9.3	8.5 to 10.0	97.90%	< 0.001
**Study location**							
Europe	59	3607	33,673	11.7	10.5 to 13.0	92.70%	< 0.001
Asia	16	5329	86,333	4.5	3.1 to 5.9	98.60%	< 0.001
North America	89	10,115	131,564	7.7	6.9 to 8.6	97.60%	< 0.001
Oceania	4	1895	16,292	16.2	5.9 to 26.5	99.10%	< 0.001
**Study period**							
1976–1999	80	4851	59,816	8.6	7.7 to 9.5	95.00%	< 0.001
2000–2009	63	5612	78,018	8.6	7.5 to 9.6	97.20%	< 0.001
2010–2019	25	10,483	130,028	9.9	8.4 to 11.4	99.10%	< 0.001
**CPR type**							
Bystander CPR	35	4493	39,974	11.3	9.3 to 13.2	97.40%	< 0.001
EMS CPR	27	1754	14,108	10.7	8.2 to 13.2	95.80%	< 0.001
Unspecific CPR	106	14,699	213,780	7.7	7.0 to 8.3	97.40%	< 0.001
**Origin of OHCA**							
Cardiac etiology	84	11,765	132,292	10.0	9.1 to 10.9	97.00%	< 0.001
Non-traumatic	62	7117	111,171	7.0	6.2 to 7.9	97.70%	< 0.001
All patients	13	879	9826	8.3	5.4 to 11.2	96.00%	< 0.001
Others	9	1185	14,573	10.1	8.3 to 12.0	89.50%	< 0.001
**Witnessed type**							
Witnessed	44	8967	97,069	10.5	9.2 to 11.7	97.60%	< 0.001
Not witnessed	3	16	324	4.4	1.4 to 7.4	40.00%	0.189
Mixed	118	11,951	170,341	8.2	7.5 to 8.9	97.20%	< 0.001
Unspecific events	3	12	128	8.9	4.0 to 13.8	0.00%	0.705
**One-month survival**^§^	**54**	**247,999**	**2,362,223**	**10.7**	**9.1 to 12.3**	**99.9%**	**< 0.001**
**Sex**							
Men	4	3968	46,831	8.0	5.2 to 10.9	99.10%	< 0.001
Women	4	2477	18,891	9.5	3.2 to 15.8	99.50%	< 0.001
Combined	45	241,554	2,296,501	11.0	9.3 to 12.8	100%	< 0.001
**Study location**							
Europe	28	25,371	292,473	9.0	7.6 to 10.3	99.50%	< 0.001
Asia	21	222,285	2,066,705	12.8	10.0 to 15.5	100%	< 0.001
North America	2	41	623	6.5	4.6 to 8.5	0.00%	0.415
Oceania	3	302	2422	16.0	8.4 to 23.7	96.10%	< 0.001
**Study period**							
1976–1999	NA	NA	NA	NA	NA	NA	NA
2000–2009	28	17,304	219,965	8.0	6.7 to 9.3	99.3%	< 0.001
2010–2019	26	230,695	2,142,258	13.3	10.9 to 15.7	100%	< 0.001
**CPR type**							
Bystander CPR	25	161,386	1,074,767	12.8	9.0 to 16.7	100%	< 0.001
EMS CPR	6	37,308	666,669	12.3	8.6 to 16.0	99.70%	< 0.001
Unspecific CPR	23	49,305	620,787	7.9	7.1 to 8.7	99.20%	< 0.001
**Origin of OHCA**							
Cardiac etiology	26	21,262	208,631	10.5	9.1 to 12.0	99.10%	< 0.001
Non-traumatic	1	23	342	6.7	4.1 to 9.3	0	< 0.001
All patients	27	226,714	2,153,250	10.8	8.5 to 13.1	100%	< 0.001
Others	NA	NA	NA	NA	NA	NA	NA
**Witnessed type**							
Witnessed	27	169,542	1,055,935	13.2	10.3 to 16.1	99.9%	< 0.001
Not witnessed	NA	NA	NA	NA	NA	NA	NA
Mixed	27	78,457	1,306,288	8.3	7.4 to 9.1	99.7%	< 0.001
Unspecific events	NA	NA	NA	NA	NA	NA	NA
**One-year survival**¶	**27**	**3791**	**42,027**	**7.7**	**5.8 to 9.5**	**97.5%**	**< 0.001**
**Sex**							
Men	1	13	320	4.1	1.9 to 6.3	0.00%	< 0.001
Women	1	14	219	6.4	3.2 to 9.6	0.00%	< 0.001
Combined	25	3764	41,488	7.9	5.9 to 9.8	97.7%	< 0.001
**Study location**							
Europe	16	3378	35,604	9.2	6.4 to 12.0	98.2%	< 0.001
Asia	3	118	2504	5.3	2.7 to 8.0	87.8%	< 0.001
North America	7	96	2190	4.0	2.8 to 5.3	51.2%	0.056
Oceania	1	199	1729	11.5	10.0 to 13.0	0.0%	< 0.001
**Study period**							
1976–1999	10	407	3517	8.5	4.1 to 12.8	96.6%	< 0.001
2000–2009	14	479	7496	6.0	4.3 to 7.6	89.0%	< 0.001
2010–2019	3	2905	31,014	12.3	5.4 to 19.3	99.6%	< 0.001
**CPR type**							
Bystander CPR	2	1579	10,805	12.3	6.4 to 18.1	79.6%	0.027
EMS CPR	3	17	480	3.2	0.5 to 6.0	64.6%	0.059
Unspecific CPR	22	2195	30,742	7.6	6.1 to 9.2	95.2%	< 0.001
**Origin of OHCA**							
Cardiac etiology	25	3763	41,493	7.9	6.0 to 9.9	97.6%	< 0.001
Non-traumatic	1	24	338	7.1	4.4 to 9.8	0.0%	< 0.001
All patients	NA	NA	NA	NA	NA	NA	NA
Others	1	4	196	2.0	0 to 4.0	0.0%	< 0.001
**Witnessed type**							
Witnessed	12	283	5765	5.3	4.0 to 6.5	77.9%	< 0.001
Not witnessed	NA	NA	NA	NA	NA	NA	NA
Mixed	15	3508	36,262	9.0	6.1 to 11.9	98.3%	< 0.001
Unspecific events	NA	NA	NA	NA	NA	NA	NA

Note: *CI* confidence interval, *CPR* cardiopulmonary resuscitation, *EMS* emergency medical services, *NA* not available, *OHCA* out-of-hospital cardiac arrests, *ROSC* return of spontaneous circulation

*Two studies reported their results by study location, 2 studies reported their results by sex, 4 studies reported their results by CPR type, 2 studies reported their results by witness type, and 1 study reported their results by OHCA type; therefore, there were 74 reports from 62 studies

†Four studies reported their results by study location, 7 studies reported their results by sex, 7 studies reported their results by CPR type, 2 studies reported their results by witness type, and 1 study reported their results by OHCA type; therefore, there were 122 reports from 88 studies

^**‡**^Three studies reported their results by study location, 21 studies reported their results by sex, 16 studies reported their results by CPR type, 1 study reported their results by witness type, and 2 studies reported their results by OHCA type; therefore, there were 168 reports from 103 studies

^§^Four studies reported their results by sex, 4 studies reported their results by CPR type, 2 studies reported their results by OHCA type, and 1 study reported their results by year; therefore, there were 54 reports from 33 studies

¶One study reported their results by sex, 2 studies reported their results by CPR type, and 1 study reported their results by witness type; therefore, there were 27 reports from 22 studies

### Survival to admission

A total of 88 studies with 122 reports estimated the incidence of survival to admission of OHCA patients who underwent CPR. From the random-effects meta-analysis, an incidence of 22.0% (95% CI 20.7–23.4%) for survival to admission was estimated globally among OHCA patients who received CPR.

With regard to study location, Oceania had the highest incidence (33.5%; 95% CI 21.7–45.3%), followed by Europe (25.7%; 95% CI 23.9–27.6%), North America (20.5%; 95% CI 18.1–22.9%), and Asian countries (15.6%; 95% CI 13.2–18.0%).

### Survival to discharge

One hundred and three studies with 168 reports investigated the rate of survival to discharge of OHCA patients who underwent CPR. From the random-effects meta-analysis, an incidence of 8.8% (95% CI 8.2–9.4%) for survival to discharge was estimated globally among OHCA patients, and there was high heterogeneity across studies (*P* < 0.001; *I*^*2*^ = 97.6%) (Table 1). The rate of survival to discharge of OHCA patients who received CPR increased from 8.6% (95% CI 7.7–9.5%) in 1976–1999 to 9.9% (95% CI 8.4–11.4%) in 2010–2019.

Subgroup analyses showed significant differences in the survival rate by study location and provider of CPR. Across the study locations, Oceania had the highest survival rate (16.2%; 95% CI 5.9–26.5%), followed by Europe (11.7%; 95% CI 10.5–13.0%), North America (7.7%; 95% CI 6.9–8.6%), and Asia (4.5%; 95% CI 3.1–5.9%). Regarding the provider of CPR, the survival rate was relatively higher among patients who received bystander CPR (11.3%; 95% CI 9.3–13.2%).

### One-month survival rate

In total, 33 studies with 54 reports investigated the 1 month survival rate of OHCA patients who underwent CPR. From the random-effects meta-analysis, an incidence of 10.7% (95% CI 9.1–12.3%) for 1-month survival was estimated globally among OHCA patients who had received CPR, and there was high heterogeneity among the studies (*P* < 0.001; *I*^*2*^ = 99.9%).

Subgroup analyses showed significant differences in 1-month survival by study location and study period. Across the study locations, Oceania had the highest survival rate (16.0%; 95% CI 8.4–23.7%), followed by Asia (12.8%; 95% CI 10.0–15.5%), Europe (9.0%; 95% CI 7.6–10.3%), and North America (6.5%; 95% CI 4.6–8.5%). Across the study periods, the 1-month survival rate increased from 8.0% (95% CI, 6.7–9.3%) in 2000–2009 to 13.3% (95% CI 10.9–15.7%) in 2010–2019.

### One-year survival rate

Twenty-seven reports from 22 studies investigated the 1 year survival rate of OHCA patients who underwent CPR. From the random-effects meta-analysis, an incidence of 7.7% (95% CI 5.8–9.5%) for 1-year survival was estimated globally among OHCA patients who received CPR, and there was high heterogeneity among the studies (*P* < 0.001; *I*^*2*^ = 97.5%).

Subgroup analyses showed significant differences in 1-year survival by study location and study period. Across the study locations, Oceania had the highest survival rate (11.5%; 95% CI 10.0–13.0%), followed by Europe (9.2%; 95% CI 6.4–12.0%), Asia (5.3%; 95% CI 2.7–8.0%), and North America (4.0%; 95% CI 2.8–5.3%). Across the study periods, the 1-year survival rate increased from 6.0% (95% CI 4.3–7.6%) in 2000–2009 to 12.3% (95% CI 5.4–19.3%) in 2010–2019. Regarding the provider of CPR, the survival rate was relatively higher among patients who received bystander CPR (12.3%; 95% CI 6.4–18.1%).

### Sensitivity analyses

The exclusion of studies with sample sizes less than 100 yielded pooled incidences of 29.0% (95% CI 26.8–31.2%, *P* < 0.001), 21.6% (95% CI 20.1–23.0%, *P* < 0.001), 8.6% (95% CI 8.0–9.2%, *P* < 0.001), 10.8% (95% CI 9.2–12.4%, *P* < 0.001), and 7.6% (95% CI 5.7–9.5%, *P* < 0.001) for ROSC, survival to hospital admission, survival to hospital discharge, 1-month survival, and 1-year survival, respectively. Thus, the survival rate did not change significantly when the observations with sample sizes less than 100 were excluded, which indicated that studies with small sample sizes did not influence the overall result. Furthermore, after the exclusion of these studies (*n* = 19) including pediatric patients, the pooled survival rates for ROSC, survival to hospital admission, survival to hospital discharge, 1-month survival, and 1-year survival were 30.5% (95% CI 26.7–34.3%, *P* < 0.001), 21.8% (95% CI 20.4–23.3%, *P* < 0.001), 9.7% (95% CI 8.9–10.5%, *P* < 0.001), 10.1% (95% CI 7.9–12.3%, *P* < 0.001), and 7.6% (95% CI 5.6–9.6%, *P* < 0.001), respectively.

Sensitivity analyses were performed by omitting each study in turn and combining the results of the remaining included studies. The overall summary survival rates for ROSC, survival to admission, survival to discharge, and the 1-month and 1-year survival rates did not alter substantially. The pooled survival rates derived from the sensitivity analyses for ROSC ranged from 28.8% (95% CI 27.5–30.1%) to 30.3% (95% CI 27.6–33.0%), those for survival to hospital admission ranged from 21.7% (95% CI 20.4–23.1%) to 22.2% (95% CI 20.8–23.7%), those for survival to hospital discharge ranged from 8.6% (95% CI 8.0–9.2%) to 8.9% (95% CI 8.2–9.5%), those for 1-month survival ranged from 10.1% (95% CI 8.5–11.7%) to 10.9% (95% CI 9.3–12.4%), and those for 1-year survival ranged from 6.9% (95% CI 5.2–8.6%) to 7.9% (95% CI 6.0–9.8%).

### Publication bias

**Fig. 2:**
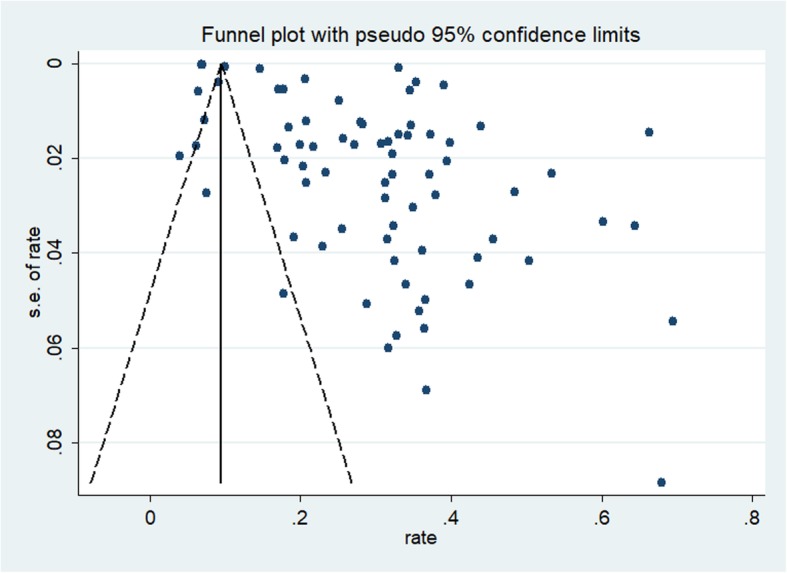
Funnel plot for the incidence of ROSC among OHCA patients who underwent CPR

**Fig. 3:**
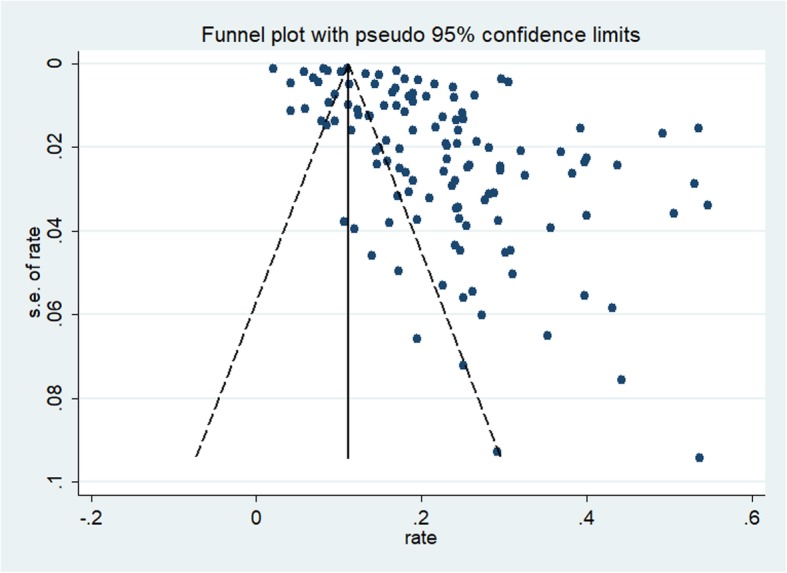
Funnel plot for the incidence of survival to admission among OHCA patients who underwent CPR

**Fig. 4:**
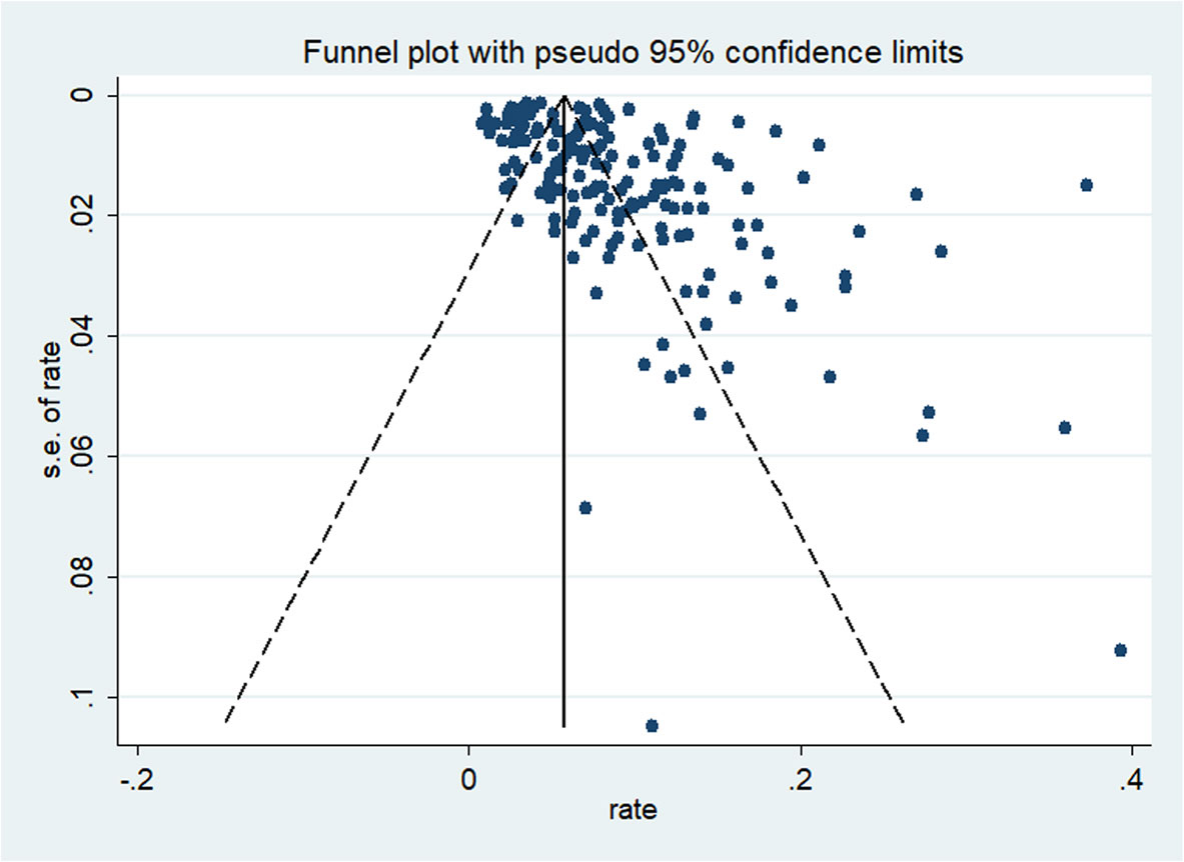
Funnel plot for the incidence of survival to discharge among OHCA patients who underwent CPR

**Fig. 5:**
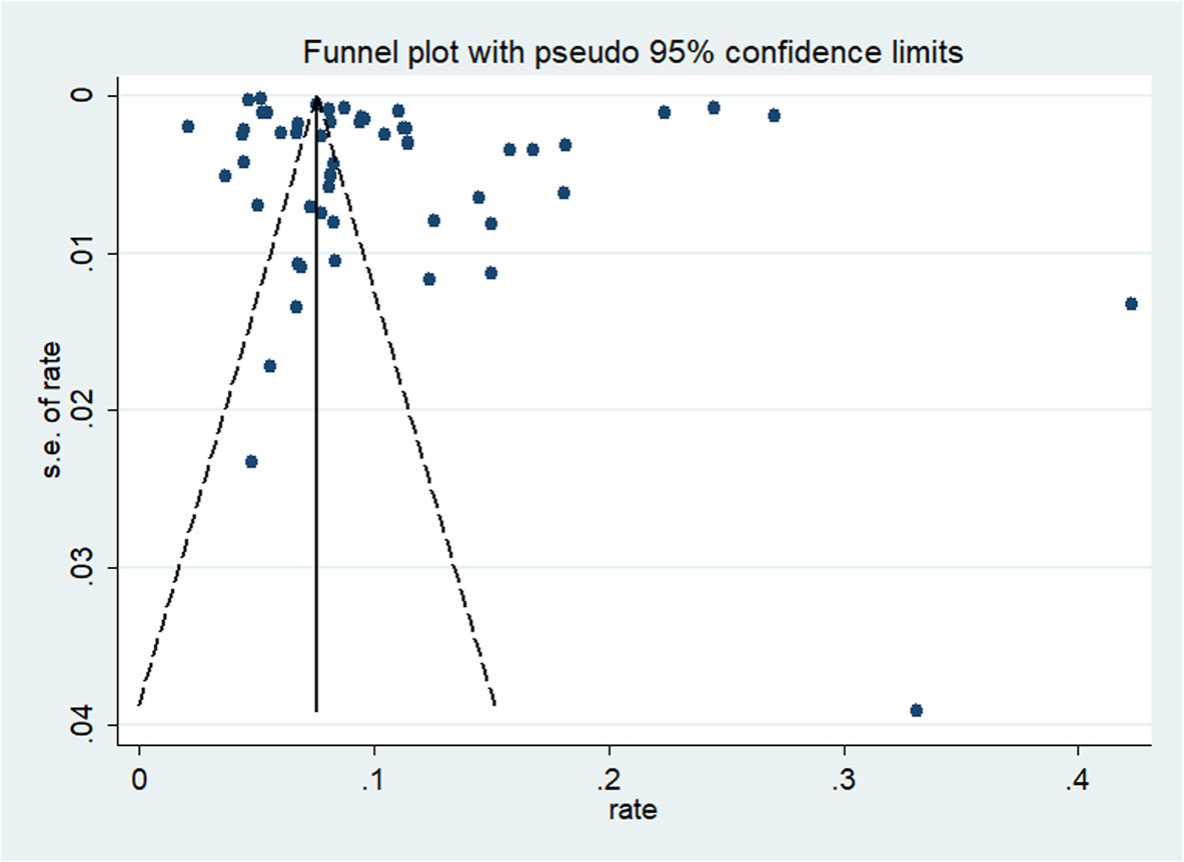
Funnel plot for the 1 month survival rate among OHCA patients who underwent CPR

**Fig. 6:**
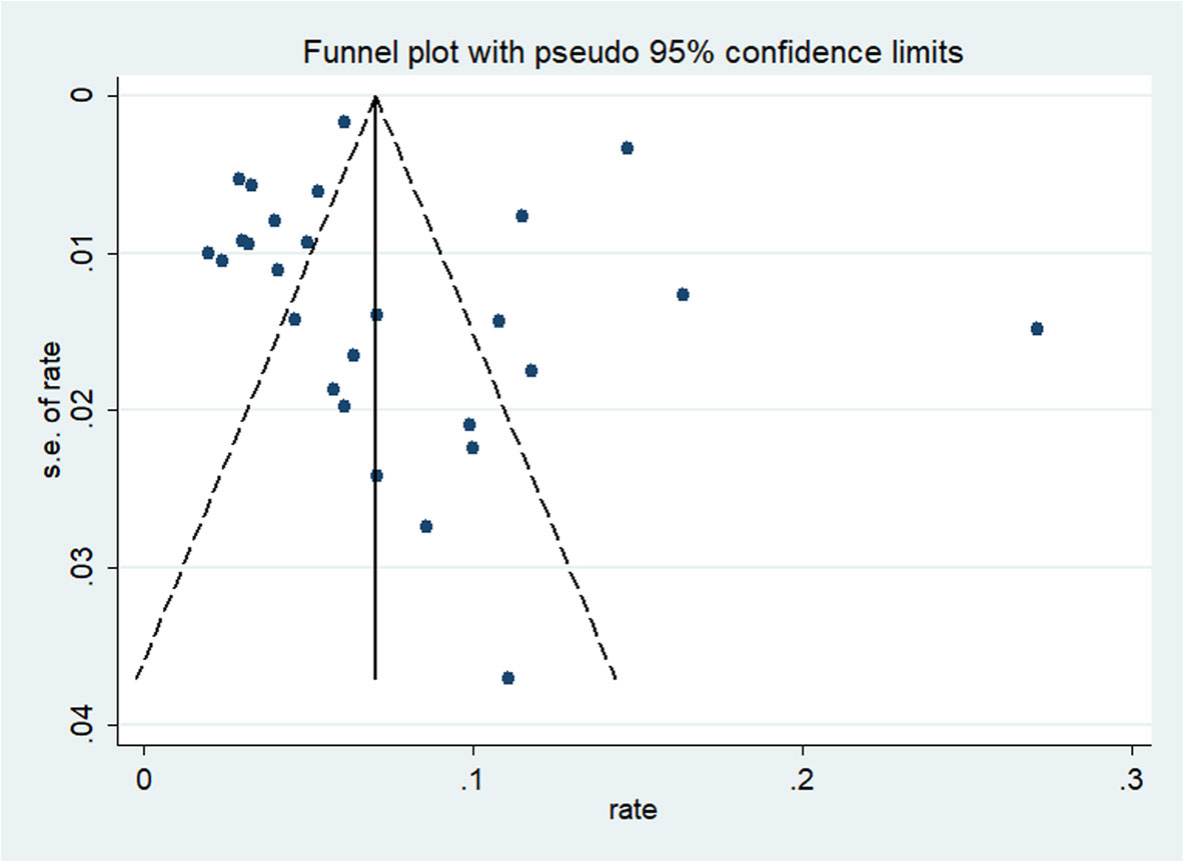
Funnel plot for the 1 year survival rate among OHCA patients who underwent CPR

The funnel plot was symmetrical for the meta-analysis of the incidence of ROSC, survival to hospital admission, survival to hospital discharge, 1-month survival, and 1-year survival in OHCA patients who received CPR (see Figs. 2, 3, 4, 5, and 6). The Egger’s test revealed no evidence of publication bias among the studies that reported the incidence of ROSC, survival to admission, survival to hospital discharge, and 1-year survival rate (Egger’s *P* = 0.362 for ROSC; Egger’s *P* = 0.128 for survival to admission; Egger’s *P* = 0.112 for survival to hospital discharge; Egger’s *P* = 0.168 for 1-year survival). However, for the 1-month survival among OHCA patients who underwent CPR, we found that Egger’s test revealed evidence of publication bias across studies (Egger’s *P* < 0.05).

## Discussion

This is the first comprehensive systematic review and meta-analysis bringing together 40 years of research to estimate the incidence of ROSC, rate of survival to admission, rate of survival to discharge, 1-month survival rate, and 1-year survival rate among OHCA patients who received CPR worldwide. We found that the pooled incidence of ROSC, and survival to admission, survival to discharge, 1-month survival, and 1-year survival rates were 29.7%, 22.0%, 8.8%, 10.7%, and 7.7%, respectively. In addition, much lower rates of the incidence of ROSC, survival to admission, and survival to discharge were observed across Asian countries and much higher survival to discharge, 1-month survival, and 1-year survival rates were found among OHCA patients who had received bystander CPR. Finally, survival to discharge among OHCA patients who underwent CPR significantly improved over the 40-year period.

Two previous studies published in 2010 [19] and 2013 [20] investigated the survival rate of OHCA patients who received CPR. The results of this current meta-analysis generally concur and further complement the findings of a previous review in several important aspects. Van de Glind et al. [20] reported that the pooled survival to discharge among patients > 70 years of age was 4.1% (95% CI 3.0–5.6%), which was lower than that in the present study (8.8%: 95% CI 8.2–9.4%). One possible reason was that there were differences in the study population and sample size. The review by Van de Glind and colleagues included only 23 studies among older patients aged > 70 years. However, our study population included all age groups. Several studies showed that increasing age was significantly associated with worse survival [21–23]. Additionally, their review did not fully investigate other subgroups or perform sensitivity analyses. Sasson et al. [19] found that the pooled survival to hospital discharge rate was 7.6% (95% CI 6.7–8.4%). However, 62 studies were not included in their analysis because their search time was limited to 2008, which may have led to overestimation of the survival rate. In addition, our review performed more detailed subgroup analyses (stratified by sex, study location, study period, type of OHCA, type of CPR, and type of cardiac arrest witness) to test the robustness of the results and explore the potential heterogeneity.

Our subgroup analyses led to two valuable findings. First, much lower rates of ROSC, survival to admission, and survival to discharge were observed in Asian countries than in European counties. These observed differences may in part reflect the differences in first registered arrhythmia as VT/VF, witnessed collapse, bystander CPR, and early defibrillation in various countries [4, 24]. These factors were significantly associated with the survival rates of OHCA patients [25–27]. Another explanation is that compared with North America and Europe, and the popularization of bystander CPR has been relatively delayed in Asia; thus, the quality of bystander CPR might be lower in Asia. In addition, a previous study showed that the thresholds of EMS protocols for initiating resuscitation are lower in Asian countries, [1] which was likely to contribute to the differences in the survival rates.

We also found much higher rates of survival to discharge, 1 month survival, and 1 year survival among OHCA patients who received bystander CPR, which was consistent with the findings of previous studies [26, 28–30]. This suggests that efforts, such as targeted CPR training to increase the bystander CPR rate, will have a substantial effect on improving the survival rates after OHCA [6, 31]. Thus, facilitating bystander CPR training is an important and effective measure that governments worldwide can implement to improve the outcome of OHCA patients.

### Strengths and limitations

The present study has several strengths. First, this is the first study to date investigating the survival of OHCA patients worldwide. Second, based on the subgroup analysis, we showed that the survival to discharge rate was much lower in developing countries than in developed counties, and the ROSC and survival to discharge rates were decreasing. Finally, as the incidence of OHCA is increasing in modern society, the results of our study can not only serve as baseline data for the global assessment of OHCA prevention interventions (evidence-based region-specific guideline updates of CPR for OHCA) but also provide a reference for international comparisons.

Potential limitations in this study need to be acknowledged. A high degree of heterogeneity was observed in this meta-analysis. The heterogeneity across studies may result from differences in the EMS system, research method, samples, provider and quality of CPR (e.g., bystander CPR, EMS CPR), and Utstein definition. However, the sensitivity analyses and consistent results from various subgroup analyses suggested that the estimates were relatively robust, and the heterogeneity can be overestimated when studies with large sample sizes are pooled. Second, only those studies published in English were included in this meta-analysis and studies in other languages were omitted. Third, the literature searches were carried out in three databases, which may be considered a source of bias.

### Suggestions for further research

More effort should be put into future research. First, more studies should be included to explore the predictors of survival of OHCA and investigate the associations between survival after OHCA and the predictors (age, sex, location of cardiac arrest, response time, CPR by bystander or EMS-physician-guided CPR, AED utilization). This would help elucidate the reasons for improved survival and the underlying mechanisms. Second, investigating the quality of life and cognitive and functional changes in survivors after OHCA will be valuable. Finally, more studies validating the cost-effectiveness of bystander CPR training or AED utilization are warranted.

## Conclusion

In conclusion, this meta-analysis suggests that the rates of survival to discharge, 1 month survival, and 1 year survival are increasing among OHCA patients who receive CPR globally. Relatively lower survival to discharge rates were observed in Asian countries. Higher rates of survival to discharge, 1-month survival, and 1-year survival were found among OHCA patients who had their cardiac arrest witnessed by EMS or a bystander and who received bystander CPR.

## Supplementary information

Additional file 1: **Table S1.** Characteristics of included studies. Supplementary references.

## Data Availability

Data may be made available by contacting the corresponding author.

## Abbreviations

AED: Automated external defibrillator
CI: Confidence interval
CPR: Cardiopulmonary resuscitation
EMS: Emergency medical services
OHCA: Out-of-hospital cardiac arrests
ROSC: Return of spontaneous circulation
