# Supplementary material for: The global survival rate among adult out-of-hospital cardiac arrest patients who received cardiopulmonary resuscitation: a systematic review and meta-analysis
Source: Crit Care. 2020 Feb 22;24:61. doi: 10.1186/s13054-020-2773-2 (PMC7036236; doi:10.1186/s13054-020-2773-2)
Supplement: Supplementary file 1 — Table S1. Characteristics of included studies. Supplementary references. [file 13054_2020_2773_MOESM1_ESM.doc]

**Appendix**

**Supplement table 1. Characteristics of included studies**

**Supplementary references**

**Supplemental Table 1. Characteristics of included studies**

| **No** | **Author** | **Year** | **Location** | **Age, years** | **Origin of OHCA** | **CPR type** | **Witnessed** | **Outcomes** |
| --- | --- | --- | --- | --- | --- | --- | --- | --- |
| 1 | Rajan et al | 2016 | Denmark | median: 66 to 75 | Cardiac etiology | Bystander CPR | wit, unwit | ROSC, 1-month survival |
| 2 | Wissenberg et al | 2013 | Denmark | median: 72 | Cardiac etiology | CPR | wit, unwit | survival to admission, 1-month survival, one-year survival |
| 3 | Holmberg et al | 2005 | Sweden | 67.7±17.0/68.2±16.3 | All patients | CPR | wit, unwit | 1-month survival |
| 4 | Iwami et al | 2015 | Japan | median: 76/79 | All patients | Bystander CPR | wit, unwit | ROSC, 1-month survival |
| 5 | Hasselqvist-Ax et al | 2015 | Sweden | median: 74/69 | Cardiac, all patients | Bystander CPR | wit | 1-month survival |
| 6 | Holmberg et al | 2001 | Sweden | median: 71 | Cardiac etiology | Bystander CPR, CPR | wit, unwit | survival to admission, 1-month survival |
| 7 | Kitamura et al | 2012 | Japan | mean: 72.3 | All patients | Bystander CPR, CPR | wit | ROSC, 1-month survival |
| 8 | Bohm et al | 2007 | Sweden | 63±18/66±16 | All patients | Bystander CPR | wit, unwit | survival to admission, 1-month survival |
| 9 | Van Hoeyweghen et al | 1993 | Belgium | NA | Cardiac etiology | Bystander CPR | NA | survival to admission |
| 10 | Svensson et al | 2010 | Sweden | mean: 68/67 | Cardiac etiology | Bystander CPR | wit | survival to discharge, 1-month survival |
| 11 | Holmberg et al | 2000 | Sweden | median: 67/73 | Cardiac etiology | Bystander CPR, CPR | wit, unwit | 1-month survival |
| 12 | Herlitz et al | 2005 | Sweden | 70±15/62±18/66±17 | All patients | Bystander CPR | wit, unwit | survival to admission, 1-month survival |
| 13 | Hollnberg et al | 2008 | Sweden | median: 71 to 72 | All patients | CPR | wit, unwit | survival to admission, 1-month survival |
| 14 | Ogawa et al | 2011 | Japan | mean: 73.1/72.7 | All patients | Bystander CPR | wit | 1-month survival |
| 15 | Ong et al | 2015 | Japan, Korea, Malaysia, Singapore, Thailand, Taiwan, UAE | mean: 49.7 to 71.7 | Cardiac etiology | Bystander CPR | wit | survival to admission, survival to discharge |
| 16 | Kaneko et al | 2017 | Japan | median: 75 to 77 | All patients | Bystander CPR | wit | ROSC, 1-month survival |
| 17 | May et al US | 2018 | US | 60.4±20.5 | Non-traumatic | CPR, Bystander CPR, EMS CPR | wit, unwit | survival to admission, survival to discharge |
| 18 | Ro et al | 2015 | Japan, Korea | median: 67/76 | Cardiac etiology | CPR | wit | survival to admission, survival to discharge |
| 19 | Kim et al | 2018 | Korea | median: 69 | Cardiac etiology | CPR | wit, unwit | survival to admission, survival to discharge |
| 20 | Iwami et al | 2007 | Japan | 70.0±15.0/68.2±15.3/69.1±16.1 | Cardiac etiology | Bystander CPR, CPR | wit | ROSC, survival to admission, 1-month survival, 1-year survival |
| 21 | Ong et al | 2008 | Singapore | 58.6±15.8/56.0±20.1 | All patients | Bystander CPR | wit, unwit | ROSC, survival to admission, survival to discharge |
| 22 | Olasveengen et al | 2008 | Norway | 65±17/62±18/63±18 | Non-traumatic | Bystander CPR | wit, unwit | ROSC, survival to admission, survival to discharge |
| 23 | Iwami et al | 2012 | Japan | 61.3±16.2/61.3±19.1 | Cardiac etiology | Bystander CPR | wit | ROSC, 1-month survival |
| 24 | SOS-KANTO Study Group | 2007 | Japan | median: 68 | All patients | Bystander CPR | wit | 1-month survival |
| 25 | Waalewijn et al | 2001 | Netherlands | NA | NA | Bystander CPR | wit | survival to admission, survival to discharge |
| 26 | Kitamura et al | 2014 | Japan | 79.4±8.6 | Cardiac etiology | CPR | wit | ROSC, survival to admission, 1-month survival |
| 27 | HAllstrom et al | 2000 | US | mean: 68 | Cardiac etiology | Bystander CPR | wit, unwit | survival to admission, survival to discharge |
| 28 | Rea et al | 2010 | US | 63.4±16.5/63.9±16.3 | All patients | Bystander CPR | wit, unwit | survival to discharge |
| 29 | Lund et al | 1976 | Norway | NA | Cardiac etiology | Bystander CPR, EMS CPR | wit, unwit | survival to discharge |
| 30 | Bobrow et al | 2010 | US | 65.3±15.2/median: 66 | Cardiac etiology | Bystander CPR, CPR | wit, unwit | survival to discharge |
| 31 | GAllagher et al | 1995 | US | NA | Cardiac etiology | Bystander CPR | wit, unwit | survival to discharge |
| 32 | Cummins et al | 1985 | US | 63±12/66±12 | Cardiac etiology | Bystander CPR | wit | survival to discharge |
| 33 | Mathiesen et al | 2018 | Norway | median: 69/70 | Cardiac etiology | Bystander CPR | wit, unwit | survival to discharge |
| 34 | Nehmea et al | 2016 | Australia | median: 73 | Cardiac etiology | EMS CPR | wit | ROSC, survival to admission, survival to discharge |
| 35 | Lindner et al | 2011 | Norway | 70±15 | Cardiac etiology | CPR | wit, unwit | ROSC, survival to discharge, 1-year survival |
| 36 | Fothergill et al | 2013 | UK | 64±16.7/66±16.0/64±15.9/64±17.1/65±16.7 | Cardiac etiology | CPR | wit | survival to discharge |
| 37 | Blewer et al | 2018 | US and Canada | 64±17 | Non-traumatic | Bystander CPR | wit, unwit | ROSC, survival to discharge |
| 38 | Shao et al | 2014 | China | 65.3±14.0 | Cardiac etiology | Bystander CPR, CPR | wit, unwit | ROSC, survival to admission, survival to discharge |
| 39 | Steinmetz et al | 2008 | Denmark | median: 67/68 | Cardiac, All patients | CPR | wit, unwit | ROSC, survival to discharge, 1-month survival |
| 40 | Nichol et al | 2008 | US, Canada | median: 67 | Non-traumatic | CPR | wit, unwit | survival to discharge |
| 41 | Westfal et al | 1996 | US | median: 68.5 | Cardiac etiology | Bystander CPR, CPR | wit | ROSC, survival to admission, survival to discharge |
| 42 | Eckstein et al | 2005 | US | 67±17 | Non-traumatic | CPR | wit, unwit | ROSC, survival to discharge |
| 43 | Cheung et al | 2006 | Australia | median: 70 | Cardiac etiology | CPR | wit, unwit | 1-month survival, 1-year survival |
| 44 | Ahna et al | 2010 | Korea | ≥0 year | Non-traumatic | CPR | wit, unwit | ROSC, survival to admission, survival to discharge |
| 45 | Weston et al | 1997 | UK | NA | Cardiac etiology | CPR | wit, unwit | survival to admission, survival to discharge |
| 46 | Finn et al | 2001 | Australia | 65.1±15.1 | Cardiac etiology | EMS CPR, Bystander CPR | wit | ROSC, 1-month survival |
| 47 | Ritter et al | 1985 | US | NA | NA | Bystander CPR, EMS CPR, CPR | wit, unwit | survival to admission, survival to discharge |
| 48 | Adielsson et al | 2011 | Sweden | 69±12/median: 71 | Cardiac etiology | Bystander CPR | wit | survival to admission, 1-month survival |
| 49 | Herlitz et al | 2003 | Sweden | 65±16/68±14/median: 69/71 | All patients | CPR | wit, unwit | survival to admission, survival to discharge |
| 50 | Lai et al | 2015 | Singapore | 60.6±19.3/63.5±18.2/median: 63.4/65.0 | Cardiac etiology | CPR | wit, unwit | survival to admission |
| 51 | Tanaka et al | 2017 | Japan, Korea, Malaysia, Singapore, Thailand, Taiwan, Emirates | 72.7±15.9/median: 76.0 | Non-traumatic | CPR | wit, unwit | survival to admission |
| 52 | Swor et al | 1995 | US | 65.9±15.3 | Cardiac etiology | Bystander CPR | wit | survival to admission, survival to discharge |
| 53 | Stiell et al | 1999 | Canada | 68±13.9 | Cardiac etiology | CPR, Bystander CPR | wit, unwit | ROSC, survival to admission, survival to discharge |
| 54 | Gaieski et al | 2017 | US | median: 64 | Non-traumatic | CPR | wit, unwit | ROSC |
| 55 | Nehme et al | 2015 | Australia | median: 70 | Cardiac etiology | CPR | wit, unwit | ROSC, survival to admission, survival to discharge |
| 56 | Herlitz et al | 2008 | Sweden | 67.2±17.5 | All patients | CPR, Bystander CPR | wit, unwit | survival to admission, 1-month survival |
| 57 | Sondergaard et al | 2018 | Denmark | median: 67/70/74 | Cardiac etiology | Bystander CPR | wit, unwit | 1-month survival, 1-year survival |
| 58 | Swor et al | 2000 | US | 66.5±15.3 | Cardiac etiology | CPR | wit, unwit | survival to admission, survival to discharge |
| 59 | Groh et al | 2001 | US | 59.0±19.4/66.4±15.0 | Non-traumatic | CPR, Bystander CPR | wit, unwit | survival to admission, survival to discharge |
| 60 | Wilson et al | 1984 | US | NA | Non-traumatic | Bystander CPR, EMS CPR, CPR | wit, unwit | survival to admission, survival to discharge |
| 61 | Smith et al | 1985 | US | NA | Non-traumatic | CPR | wit, unwit | survival to admission, survival to discharge |
| 62 | Aprahamian et al | 1986 | US | 63±18 | Non-traumatic | CPR | wit, unwit | survival to admission, survival to discharge |
| 63 | Bachman et al | 1986 | US | mean age: 65.2 | Cardiac etiology | EMS CPR | wit | survival to admission, survival to discharge |
| 64 | Bonnin et al | 1989 | US | mean age: 71 | Non-traumatic | CPR | wit, unwit | survival to admission, survival to discharge |
| 65 | Becker et al | 1991 | US | 67±16 | Non-traumatic | CPR, Bystander CPR | wit | survival to admission, survival to discharge |
| 66 | Brison et al | 1992 | Canada | 68.1±12.4 | Cardiac etiology | CPR, Bystander CPR | wit | survival to admission, survival to discharge |
| 67 | Bonnin et al | 1993 | US | 65±14 | Non-traumatic | CPR, Bystander CPR | wit, unwit | ROSC, survival to discharge |
| 68 | Kellermann et al | 1993 | US | mean: 63.5/64.6 | Cardiac etiology | CPR, Bystander CPR | wit | ROSC, survival to admission, survival to discharge |
| 69 | Pepe et al | 1993 | US | 65±14 | Cardiac etiology | CPR | wit | survival to discharge |
| 70 | Richless et al | 1993 | US | 67.3±12.3 | Cardiac etiology | EMS CPR | wit, unwit | survival to admission, survival to discharge |
| 71 | Tresch et al | 1993 | US | 78.5±11.8 | NA | EMS CPR | wit, unwit | survival to admission, survival to discharge, 1-year survival |
| 72 | Van Hoeven et al | 1993 | Netherlands | 61.7±15.3 | Non-traumatic | Bystander CPR, CPR | wit, unwit | survival to admission, survival to discharge |
| 73 | Kass et al | 1994 | US | NA | Cardiac etiology | CPR | wit | ROSC, survival to admission, survival to discharge, 1-year survival |
| 74 | Lombardi et al | 1994 | US | median: 70 | Cardiac etiology | CPR, Bystander CPR, EMS CPR | wit | ROSC, survival to admission, survival to discharge |
| 75 | Schneider et al | 1994 | Germany | 63.2±16.4 | Cardiac etiology | CPR | wit, unwit | survival to admission, survival to discharge |
| 76 | Crone et al | 1995 | Auckland | NA | Cardiac etiology | CPR | wit | survival to admission, survival to discharge |
| 77 | Hodgetts et al | 1995 | UK | NA | Non-traumatic | EMS CPR | wit, unwit | ROSC, survival to discharge |
| 78 | Rainer et al | 1995 | UK | median: 66/62 | Non-traumatic | CPR | wit, unwit | survival to admission, survival to discharge |
| 79 | Giraud et al | 1996 | France | mean: 58 | Cardiac etiology | CPR | wit | ROSC, survival to admission, survival to discharge, 1-year survival |
| 80 | Killien et al | 1996 | US | mean: 66 | Cardiac etiology | CPR | wit, unwit | survival to admission, survival to discharge |
| 81 | Kuisma et al | 1996 | Finland | 62.2±18.4 | Cardiac etiology | CPR | wit | ROSC, survival to admission, survival to discharge |
| 82 | Adams et al | 1997 | UK | mean age: 67 | NA | CPR | wit | survival to discharge |
| 83 | Fischer et al | 1997 | Germany | NA | Cardiac etiology | CPR, EMS CPR | wit, unwit | ROSC, survival to admission, survival to discharge, 1-year survival |
| 84 | Kuisma et al | 1997 | Finland | 56.7±21.2 | Cardiac etiology, All patients | Bystander CPR, CPR | unwit | ROSC, survival to admission, survival to discharge |
| 85 | Mitchell et al | 1997 | UK | mean: 67/69 | Cardiac etiology | EMS CPR | wit | ROSC, survival to admission, survival to discharge |
| 86 | Stapczynski et al | 1997 | US | 65±17/67±16 | Non-traumatic | CPR, Bystander CPR | wit, unwit | survival to admission, survival to discharge |
| 87 | Valenzuela et al | 1997 | US | mean: 66/64 | Non-traumatic | CPR | wit | survival to discharge |
| 88 | De vreede et al | 1998 | Netherlands | 58±11 | Non-traumatic | EMS CPR | wit, unwit | survival to discharge |
| 89 | Joyce et al | 1998 | US | mean: 66.9 | Cardiac etiology | EMS CPR | wit, unwit | survival to admission, survival to discharge |
| 90 | Kette et al | 1998 | Italy | NA | Cardiac etiology | CPR | wit | ROSC, survival to admission, survival to discharge, 1-year survival |
| 91 | Lindholm et al | 1998 | US | mean: 67 | Cardiac etiology | CPR, Bystander CPR | wit, unwit | ROSC, survival to discharge |
| 92 | Tadel et al | 1998 | Slovenia | NA | Cardiac etiology | CPR | wit, unwit | ROSC, survival to admission, survival to discharge, 1-year survival |
| 93 | Waalewijn et al | 1998 | Netherlands | mean: 64 | Cardiac etiology | CPR, Bystander CPR | wit | ROSC, survival to admission, survival to discharge |
| 94 | Absalom et al | 1999 | UK | 67±13/70±13 | Cardiac etiology | CPR | wit | ROSC, survival to admission, survival to discharge |
| 95 | Bottiger et al | 1999 | Germany | 67±12 | Cardiac etiology | CPR | wit | ROSC, survival to admission, survival to discharge, 1-year survival |
| 96 | Kuilman et al | 1999 | Netherlands | 65±13 | Cardiac etiology | CPR, Bystander CPR | wit, unwit | survival to admission, survival to discharge, 1-year survival |
| 97 | Lui et al | 1999 | China | mean: 68.7 | Non-traumatic | CPR, Bystander CPR | wit, unwit | survival to admission, survival to discharge |
| 98 | Sunde et al | 1999 | Norway | median: 69/70 | Cardiac etiology | CPR | wit | ROSC, survival to admission, survival to discharge, 1-year survival |
| 99 | Jennings and Pasco | 2001 | Australia | median: 68 | Cardiac etiology | CPR, Bystander CPR | wit, unwit | ROSC, survival to admission, survival to discharge |
| 100 | Rea et al | 2001 | US | 69.7±13.0/67.0±12.7/68.7±13.2 | Cardiac etiology | Bystander CPR | wit, unwit | survival to discharge |
| 101 | Hagihara et al | 2018 | Japan | 75.56±15.04 | Cardiac etiology | Bystander CPR | wit, unwit | ROSC, 1-month survival |
| 102 | Citerio et al | 2002 | Italy | mean: 70.2±15/median: 72 | Cardiac etiology | CPR | wit, unwit | 1-month survival |
| 103 | Fan and Leung | 2002 | Hong Kong, China | median: 73 | Non-traumatic | CPR | wit, unwit | survival to discharge |
| 104 | Lim and Seow | 2002 | Singapore | 63.1±18.3/65.5±15.9 | Non-traumatic | CPR, Bystander CPR | NA | ROSC, survival to admission, survival to discharge |
| 105 | Myerburg et al | 2002 | US | 69.5±15.1/67.8±16.3 | Non-traumatic | EMS CPR | wit, unwit | survival to discharge |
| 106 | Goto et al | 2003 | Japan | 59±4/66±2 | Cardiac etiology | CPR | wit, unwit | ROSC, 1-year survival |
| 107 | Grmec and Kupnik | 2003 | Slovenia | 57.4±13.3 | Cardiac etiology, Non-traumatic | CPR | wit, unwit | ROSC, survival to admission, survival to discharge, 1-year survival |
| 108 | Haukoos et al | 2003 | US | median: 70 | Non-traumatic | Bystander CPR, CPR | wit, unwit | survival to discharge |
| 109 | Nishiuchi et al | 2003 | Japan | 67.5±16.9 | Cardiac etiology | CPR | wit | ROSC, survival to admission, 1-month survival, 1-year survival |
| 110 | Ong et al Singopre | 2003 | Singapore | 62.2±17.9 | Cardiac etiology | CPR | wit, unwit | ROSC, survival to admission, survival to discharge |
| 111 | Horsted et al | 2004 | Denmark | median: 68 | Cardiac, All patients | Bystander CPR, CPR | wit, unwit | ROSC, survival to admission, survival to discharge, 1-year survival |
| 112 | Rudner et al | 2004 | Poland | mean: 63 | Cardiac etiology, All patients | CPR, Bystander CPR | wit, unwit | ROSC, survival to admission, survival to discharge, 1-year survival |
| 113 | Davies et al | 2005 | UK | mean: 63.4/61.2 | NA | CPR | wit, unwit | survival to discharge |
| 114 | Handel et al | 2005 | US | 65.3±17.4 | Cardiac etiology | CPR | wit, unwit | ROSC, survival to admission, survival to discharge, 1-month survival |
| 115 | Hayashi and Ujike | 2005 | Japan | mean: 67.1 | All patients | Bystander CPR, CPR | wit, unwit | ROSC, survival to admission, survival to discharge |
| 116 | White et al | 2005 | US | 66.5±14.3/63.8±13.8 | Non-traumatic | CPR | wit, unwit | survival to admission, survival to discharge |
| 117 | Drezner and Rogers | 2006 | US | mean: 21 | Cardiac etiology | CPR | wit | survival to discharge |
| 118 | Olasveengen et al | 2009 | Norway | 65±17/63±18 | Non-traumatic | EMS CPR | wit | ROSC, survival to admission, survival to discharge |
| 119 | Yen et al Taiwan | 2006 | Taiwan | 66.4±18.7/63.4±19.2 | Non-traumatic | EMS CPR | wit, unwit | survival to admission, survival to discharge |
| 120 | Ohshige et al Japan | 2005 | Japan | 67.3±17.5/69.4±18.9 | Non-traumatic | EMS CPR | wit, unwit | survival to admission, 1-month survival |
| 121 | Soo et al | 1999 | UK | median: from 65 to 71 | Cardiac etiology | EMS CPR, CPR | wit | survival to admission, survival to discharge, 1-year survival |
| 122 | Eisenburger et al | 2001 | Austria | median: 64 | Non-traumatic | CPR | wit, unwit | ROSC, survival to discharge, 1-year survival |
| 123 | Dickenson et al | 1997 | US | mean: 69.9/67.5 | Non-traumatic | EMS CPR | NA | ROSC, survival to discharge |
| 124 | Hagihara et al | 2014 | Japan | 69.6±17.2 | All patients | EMS CPR | wit, unwit | ROSC, 1-month survival |
| 125 | Yasunaga et al | 2010 | Japan | 71.6±17.8/median: 75 | All patients | CPR, EMS CPR | wit | 1-month survival |
| 126 | Hampton et al | 1977 | UK | mean: 60/61 | Cardiac etiology | EMS CPR | NA | survival to admission, survival to discharge |
| 127 | Mitchell et al | 1997 | UK, US | NA | Cardiac etiology | EMS CPR | wit, unwit | ROSC, survival to admission, survival to discharge |
| 128 | Frandsen et al | 1991 | Denmark | 66±12/68±12/69±11/66±15/55±12/62±11 | Cardiac etiology | EMS CPR | wit, unwit | survival to admission, survival to discharge |
| 129 | Fischer et al | 2011 | Germany, Spanish, UK, US | NA | Cardiac etiology | EMS CPR | NA | ROSC, survival to admission |
| 130 | Kellum et al | 2006 | US | NA | Cardiac etiology | CPR | wit, unwit | survival to discharge |
| 131 | Pleskot et al | 2006 | Czech | 67±13/median: 69 | Cardiac etiology | CPR, Bystander CPR | wit, unwit | ROSC, survival to admission, survival to discharge |
| 132 | Davis et al | 2007 | US | mean: 66.3 | Non-traumatic | CPR | wit, unwit | ROSC, survival to admission, survival to discharge |
| 133 | Daya et al | 2007 | US | ≥20 years | Non-traumatic | EMS CPR | wit | survival to discharge |
| 134 | Dunne et al | 2007 | US | 63.3±17.2 | Non-traumatic | CPR | wit, unwit | ROSC, survival to admission |
| 135 | Estner et al | 2007 | Germany | 68.1±14.6 | Cardiac etiology | CPR, Bystander CPR, EMS CPR | wit, unwit | survival to admission, survival to discharge |
| 136 | Fairbanks et al | 2007 | US | median: 67 | Cardiac etiology | CPR, Bystander CPR, EMS CPR | wit, unwit | ROSC, 1-month survival, 1-year survival |
| 137 | Herlitz et al | 2007 | Sweden | ≥0 year | Cardiac etiology, All patients | CPR, Bystander CPR | wit, unwit | 1-month survival |
| 138 | Jasinskas et al | 2007 | Lithuania | 61.64±2.73/55.64±6.94/64.39±2.84 | Cardiac etiology | EMS CPR | wit, unwit | ROSC |
| 139 | Ma et al | 2007 | Taiwan | 67.9±19.4/70.4±18.7 | Non-traumatic | Bystander CPR | wit, unwit | ROSC, survival to admission, survival to discharge |
| 140 | Morrison et al | 2007 | Canada | 69±15 | Cardiac etiology | CPR | wit | ROSC, survival to admission, survival to discharge |
| 141 | Fleischhackl et al | 2008 | Austria | median: 62.5 | All patients | CPR | wit, unwit | survival to discharge |

Note: CPR: cardiopulmonary resuscitation; EMS: emergency medical services; NA: not available; OHCA: out-of-hospital cardiac arrests; ROSC: return of spontaneous circulation.

**Supplementary References**

1. Site variation in EMS Treatment, Transport and Survival in relation to Restoration of Spontaneous Circulation (ROSC) for Adult Out-of-Hospital Cardiac Arrest The Resuscitation Outcomes Consortium (ROC) Epistry.

2. Out-of-hospital cardiac arrest. Evaluation of one year of activity in Saint-Etienne’s emergency medical system using the Utstein style.

3. Cardiopulmonary resuscitation by bystanders with chest compression only (SOS-KANTO): an observational study. *Lancet (London, England)* 2007; **369**(9565): 920-6.

4. Absalom AR, Bradley P, Soar J. Out-of-hospital cardiac arrests in an urban/rural area during 1991 and 1996: have emergency medical service changes improved outcome? *Resuscitation* 1999; **40**(1): 3-9.

5. Adams JN, Sirel J, Marsden K, Cobbe SM. Heartstart Scotland: the use of paramedic skills in out of hospital resuscitation. *Heart* 1997; **78**(4): 399-402.

6. Adielsson A, Hollenberg J, Karlsson T, et al. Increase in survival and bystander CPR in out-of-hospital shockable arrhythmia: bystander CPR and female gender are predictors of improved outcome. Experiences from Sweden in an 18-year perspective. *Heart* 2011; **97**(17): 1391-6.

7. Ahn KO, Shin SD, Suh GJ, et al. Epidemiology and outcomes from non-traumatic out-of-hospital cardiac arrest in Korea: A nationwide observational study. *Resuscitation* 2010; **81**(8): 974-81.

8. Aprahamian C, Thompson BM, Gruchow HW, et al. Decision making in prehospital sudden cardiac arrest. *Ann Emerg Med* 1986; **15**(4): 445-9.

9. Bachman JW, McDonald GS, O'Brien PC. A study of out-of-hospital cardiac arrests in northeastern Minnesota. *Jama* 1986; **256**(4): 477-83.

10. Becker LB, Ostrander MP, Barrett J, Kondos GT. Outcome of CPR in a large metropolitan area--where are the survivors? *Ann Emerg Med* 1991; **20**(4): 355-61.

11. Blewer AL, McGovern SK, Schmicker RH, et al. Gender Disparities Among Adult Recipients of Bystander Cardiopulmonary Resuscitation in the Public. *Circ Cardiovasc Qual Outcomes* 2018; **11**(8): e004710.

12. Bobrow BJ, Spaite DW, Berg RA, et al. Chest compression-only CPR by lay rescuers and survival from out-of-hospital cardiac arrest. *Jama* 2010; **304**(13): 1447-54.

13. Bohm K, Rosenqvist M, Herlitz J, Hollenberg J, Svensson L. Survival is similar after standard treatment and chest compression only in out-of-hospital bystander cardiopulmonary resuscitation. *Circulation* 2007; **116**(25): 2908-12.

14. Bonnin MJ, Pepe PE, Kimball KT, Clark PS, Jr. Distinct criteria for termination of resuscitation in the out-of-hospital setting. *Jama* 1993; **270**(12): 1457-62.

15. Bonnin MJ, Swor RA. Outcomes in unsuccessful field resuscitation attempts. *Ann Emerg Med* 1989; **18**(5): 507-12.

16. Bottiger BW, Grabner C, Bauer H, et al. Long term outcome after out-of-hospital cardiac arrest with physician staffed emergency medical services: the Utstein style applied to a midsized urban/suburban area. *Heart* 1999; **82**(6): 674-9.

17. Brison RJ, Davidson JR, Dreyer JF, et al. Cardiac arrest in Ontario: circumstances, community response, role of prehospital defibrillation and predictors of survival. *CMAJ : Canadian Medical Association journal = journal de l'Association medicale canadienne* 1992; **147**(2): 191-9.

18. Cheung W, Flynn M, Thanakrishnan G, Milliss DM, Fugaccia E. Survival after out-of-hospital cardiac arrest in Sydney, Australia. *Critical care and resuscitation : journal of the Australasian Academy of Critical Care Medicine* 2006; **8**(4): 321-7.

19. Citerio G, Galli D, Cesana GC, et al. Emergency system prospective performance evaluation for cardiac arrest in Lombardia, an Italian region. *Resuscitation* 2002; **55**(3): 247-54.

20. Crone PD. Auckland Ambulance Service cardiac arrest data 1991-3. *The New Zealand medical journal* 1995; **108**(1004): 297-9.

21. Cummins RO, Eisenberg MS, Hallstrom AP, Litwin PE. Survival of out-of-hospital cardiac arrest with early initiation of cardiopulmonary resuscitation. *Am J Emerg Med* 1985; **3**(2): 114-9.

22. Davies CS, Colquhoun MC, Boyle R, Chamberlain DA. A national programme for on-site defibrillation by lay people in selected high risk areas: initial results. *Heart* 2005; **91**(10): 1299-302.

23. Davis DP, Fisher R, Aguilar S, et al. The feasibility of a regional cardiac arrest receiving system. *Resuscitation* 2007; **74**(1): 44-51.

24. de Vreede-Swagemakers JJ, Gorgels AP, Dubois-Arbouw WI, et al. Circumstances and causes of out-of-hospital cardiac arrest in sudden death survivors. *Heart* 1998; **79**(4): 356-61.

25. Dickinson ET, Schneider RM, Verdile VP. The impact of prehospital physicians on out-of-hospital nonasystolic cardiac arrest. *Prehospital Emergency Care* 2009; **1**(3): 132-5.

26. Drezner JA, Rogers KJ. Sudden cardiac arrest in intercollegiate athletes: detailed analysis and outcomes of resuscitation in nine cases. *Heart Rhythm* 2006; **3**(7): 755-9.

27. Dunne RB, Compton S, Zalenski RJ, Swor R, Welch R, Bock BF. Outcomes from out-of-hospital cardiac arrest in Detroit. *Resuscitation* 2007; **72**(1): 59-65.

28. Eckstein M, Stratton SJ, Chan LS. Termination of resuscitative efforts for out-of-hospital cardiac arrests. *Acad Emerg Med* 2005; **12**(1): 65-70.

29. Eisenburger P, Czappek G, Sterz F, et al. Cardiac arrest patients in an alpine area during a six year period. *Resuscitation* 2001; **51**(1): 39-46.

30. Eng Hock Ong M, Chan YH, Anantharaman V, Lau ST, Lim SH, Seldrup J. Cardiac arrest and resuscitation epidemiology in Singapore (CARE I study). *Prehosp Emerg Care* 2003; **7**(4): 427-33.

31. Estner HL, Gunzel C, Ndrepepa G, et al. Outcome after out-of-hospital cardiac arrest in a physician-staffed emergency medical system according to the Utstein style. *Am Heart J* 2007; **153**(5): 792-9.

32. Fairbanks RJ, Shah MN, Lerner EB, Ilangovan K, Pennington EC, Schneider SM. Epidemiology and outcomes of out-of-hospital cardiac arrest in Rochester, New York. *Resuscitation* 2007; **72**(3): 415-24.

33. Fan KL, Leung LP. Prognosis of patients with ventricular fibrillation in out-of-hospital cardiac arrest in Hong Kong: prospective study. *Hong Kong medical journal = Xianggang yi xue za zhi* 2002; **8**(5): 318-21.

34. Finn JC, Jacobs IG, Holman CD, Oxer HF. Outcomes of out-of-hospital cardiac arrest patients in Perth, Western Australia, 1996-1999. *Resuscitation* 2001; **51**(3): 247-55.

35. Fischer M, Fischer NJ, Schuttler J. One-year survival after out-of-hospital cardiac arrest in Bonn city: outcome report according to the 'Utstein style'. *Resuscitation* 1997; **33**(3): 233-43.

36. Fischer M, Kamp J, Garcia-Castrillo Riesgo L, et al. Comparing emergency medical service systems--a project of the European Emergency Data (EED) Project. *Resuscitation* 2011; **82**(3): 285-93.

37. Fleischhackl R, Roessler B, Domanovits H, et al. Results from Austria's nationwide public access defibrillation (ANPAD) programme collected over 2 years. *Resuscitation* 2008; **77**(2): 195-200.

38. Fothergill RT, Watson LR, Chamberlain D, Virdi GK, Moore FP, Whitbread M. Increases in survival from out-of-hospital cardiac arrest: a five year study. *Resuscitation* 2013; **84**(8): 1089-92.

39. Frandsen F, Nielsen JR, Gram L, et al. Evaluation of intensified prehospital treatment in out-of-hospital cardiac arrest: survival and cerebral prognosis. The Odense ambulance study. *Cardiology* 1991; **79**(4): 256-64.

40. Gaieski DF, Agarwal AK, Abella BS, et al. Adult out-of-hospital cardiac arrest in philadelphia from 2008-2012: An epidemiological study. *Resuscitation* 2017; **115**: 17-22.

41. Gallagher EJ, Lombardi G, Gennis P. Effectiveness of bystander cardiopulmonary resuscitation and survival following out-of-hospital cardiac arrest. *Jama* 1995; **274**(24): 1922-5.

42. Goto Y, Suzuki I, Inaba H. Frequency of ventricular fibrillation as predictor of one-year survival from out-of-hospital cardiac arrests. *The American Journal of Cardiology* 2003; **92**(4): 457-9.

43. Grmec Š, Kupnik D. Does the Mainz Emergency Evaluation Scoring (MEES) in combination with capnometry (MEESc) help in the prognosis of outcome from cardiopulmonary resuscitation in a prehospital setting? *Resuscitation* 2003; **58**(1): 89-96.

44. Groh WJ, Newman MM, Beal PE, Fineberg NS, Zipes DP. Limited response to cardiac arrest by police equipped with automated external defibrillators: lack of survival benefit in suburban and rural Indiana--the police as responder automated defibrillation evaluation (PARADE). *Acad Emerg Med* 2001; **8**(4): 324-30.

45. Hagihara A, Hasegawa M, Abe T, Nagata T, Nabeshima Y. Physician presence in an ambulance car is associated with increased survival in out-of-hospital cardiac arrest: a prospective cohort analysis. *PLoS One* 2014; **9**(1): e84424.

46. Hagihara A, Onozuka D, Shibuta H, Hasegawa M, Nagata T. Dispatcher-assisted bystander cardiopulmonary resuscitation and survival in out-of-hospital cardiac arrest. *Int J Cardiol* 2018; **265**: 240-5.

47. Hallstrom A, Cobb L, Johnson E, Copass M. Cardiopulmonary resuscitation by chest compression alone or with mouth-to-mouth ventilation. *N Engl J Med* 2000; **342**(21): 1546-53.

48. Hampton JR, Dowling M, Nicholas C. Comparison of results from a cardiac ambulance manned by medical or non-medical personnel. *Lancet (London, England)* 1977; **1**(8010): 526-9.

49. Handel DA, Gallo P, Schmidt M, et al. Prehospital cardiac arrest in a paramedic first-responder system using the Utstein style. *Prehosp Emerg Care* 2005; **9**(4): 398-404.

50. Hasselqvist-Ax I, Riva G, Herlitz J, et al. Early cardiopulmonary resuscitation in out-of-hospital cardiac arrest. *N Engl J Med* 2015; **372**(24): 2307-15.

51. Haukoos JS, Lewis RJ, Stratton SJ, Niemann JT. Is the ACLS score a valid prediction rule for survival after cardiac arrest? *Acad Emerg Med* 2003; **10**(6): 621-6.

52. Hayashi H, Ujike Y. Out-of hospital cardiac arrest in Okayama city (Japan): outcome report according to the "Utsutein Style". *Acta medica Okayama* 2005; **59**(2): 49-54.

53. Herlitz J, Bang A, Gunnarsson J, et al. Factors associated with survival to hospital discharge among patients hospitalised alive after out of hospital cardiac arrest: change in outcome over 20 years in the community of Goteborg, Sweden. *Heart* 2003; **89**(1): 25-30.

54. Herlitz J, Svensson L, Engdahl J, et al. Characteristics of cardiac arrest and resuscitation by age group: an analysis from the Swedish Cardiac Arrest Registry. *Am J Emerg Med* 2007; **25**(9): 1025-31.

55. Herlitz J, Svensson L, Engdahl J, Silfverstolpe J. Characteristics and outcome in out-of-hospital cardiac arrest when patients are found in a non-shockable rhythm. *Resuscitation* 2008; **76**(1): 31-6.

56. Herlitz J, Svensson L, Holmberg S, Angquist KA, Young M. Efficacy of bystander CPR: intervention by lay people and by health care professionals. *Resuscitation* 2005; **66**(3): 291-5.

57. Hodgetts TJ, Brown T, Driscoll P, Hanson J. Pre-hospital cardiac arrest: room for improvement. *Resuscitation* 1995; **29**(1): 47-54.

58. Hollenberg J, Bang A, Lindqvist J, et al. Difference in survival after out-of-hospital cardiac arrest between the two largest cities in Sweden: a matter of time? *Journal of internal medicine* 2005; **257**(3): 247-54.

59. Hollenberg J, Herlitz J, Lindqvist J, et al. Improved survival after out-of-hospital cardiac arrest is associated with an increase in proportion of emergency crew--witnessed cases and bystander cardiopulmonary resuscitation. *Circulation* 2008; **118**(4): 389-96.

60. Holmberg M. Factors modifying the effect of bystander cardiopulmonary resuscitation on survival in out-of-hospital cardiac arrest patients in Sweden. *European Heart Journal* 2001; **22**(6): 511-9.

61. Holmberg M, Holmberg S, Herlitz J. Effect of bystander cardiopulmonary resuscitation in out-of-hospital cardiac arrest patients in Sweden. *Resuscitation* 2000; **47**(1): 59-70.

62. Horsted TI, Rasmussen LS, Lippert FK, Nielsen SL. Outcome of out-of-hospital cardiac arrest--why do physicians withhold resuscitation attempts? *Resuscitation* 2004; **63**(3): 287-93.

63. Iwami T, Kawamura T, Hiraide A, et al. Effectiveness of bystander-initiated cardiac-only resuscitation for patients with out-of-hospital cardiac arrest. *Circulation* 2007; **116**(25): 2900-7.

64. Iwami T, Kitamura T, Kawamura T, et al. Chest compression-only cardiopulmonary resuscitation for out-of-hospital cardiac arrest with public-access defibrillation: a nationwide cohort study. *Circulation* 2012; **126**(24): 2844-51.

65. Iwami T, Kitamura T, Kiyohara K, Kawamura T. Dissemination of Chest Compression-Only Cardiopulmonary Resuscitation and Survival After Out-of-Hospital Cardiac Arrest. *Circulation* 2015; **132**(5): 415-22.

66. Jasinskas N, Vaitkaitis D, Pilvinis V, Jancaityte L, Bernotiene G, Dobozinskas P. The dependence of successful resuscitation on electrocardiographically documented cardiac rhythm in case of out-of-hospital cardiac arrest. *Medicina (Kaunas, Lithuania)* 2007; **43**(10): 798-802.

67. Jennings P, Pasco J. Survival from out-of-hospital cardiac arrest in the Geelong region of Victoria, Australia. *Emergency medicine (Fremantle, WA)* 2001; **13**(3): 319-25.

68. Joyce SM, Davidson LW, Manning KW, Wolsey B, Topham R. Outcomes of sudden cardiac arrest treated with defibrillation by emergency medical technicians (EMT-Ds) or paramedics in a two-tiered urban EMS system. *Prehosp Emerg Care* 1998; **2**(1): 13-7.

69. Kaneko H, Hara M, Mizutani K, et al. Improving Outcomes of Witnessed Out-of-Hospital Cardiac Arrest After Implementation of International Liaison Committee on Resuscitation 2010 Consensus: A Nationwide Prospective Observational Population-Based Study. *J Am Heart Assoc* 2017; **6**(8).

70. Kass LE, Eitel DR, Sabulsky NK, Ogden CS, Hess DR, Peters KL. One-year survival after prehospital cardiac arrest: the Utstein style applied to a rural-suburban system. *Am J Emerg Med* 1994; **12**(1): 17-20.

71. Kellermann AL, Hackman BB, Somes G. Predicting the outcome of unsuccessful prehospital advanced cardiac life support. *Jama* 1993; **270**(12): 1433-6.

72. Kellum MJ, Kennedy KW, Ewy GA. Cardiocerebral resuscitation improves survival of patients with out-of-hospital cardiac arrest. *Am J Med* 2006; **119**(4): 335-40.

73. Kette F, Sbrojavacca R, Rellini G, et al. Epidemiology and survival rate of out-of-hospital cardiac arrest in north-east Italy: The F.A.C.S. study. Friuli Venezia Giulia Cardiac Arrest Cooperative Study. *Resuscitation* 1998; **36**(3): 153-9.

74. Killien SY, Geyman JP, Gossom JB, Gimlett D. Out-of-hospital cardiac arrest in a rural area: a 16-year experience with lessons learned and national comparisons. *Ann Emerg Med* 1996; **28**(3): 294-300.

75. Kim JY, Hwang SO, Shin SD, et al. Korean Cardiac Arrest Research Consortium (KoCARC): rationale, development, and implementation. *Clin Exp Emerg Med* 2018; **5**(3): 165-76.

76. Kitamura T, Iwami T, Kawamura T, et al. Nationwide improvements in survival from out-of-hospital cardiac arrest in Japan. *Circulation* 2012; **126**(24): 2834-43.

77. Kitamura T, Morita S, Kiyohara K, et al. Trends in survival among elderly patients with out-of-hospital cardiac arrest: a prospective, population-based observation from 1999 to 2011 in Osaka. *Resuscitation* 2014; **85**(11): 1432-8.

78. Kuilman M, Bleeker JK, Hartman JA, Simoons ML. Long-term survival after out-of-hospital cardiac arrest: an 8-year follow-up. *Resuscitation* 1999; **41**(1): 25-31.

79. Kuisma M, Jaara K. Unwitnessed out-of-hospital cardiac arrest: is resuscitation worthwhile? *Ann Emerg Med* 1997; **30**(1): 69-75.

80. Kuisma M, Maatta T. Out-of-hospital cardiac arrests in Helsinki: Utstein style reporting. *Heart* 1996; **76**(1): 18-23.

81. Lai H, Choong CV, Fook-Chong S, et al. Interventional strategies associated with improvements in survival for out-of-hospital cardiac arrests in Singapore over 10 years. *Resuscitation* 2015; **89**: 155-61.

82. Lim GH, Seow E. Resuscitation for Patients with Out-of-Hospital Cardiac Arrest: Singapore. *Prehospital and Disaster Medicine* 2012; **17**(02): 96-101.

83. Lindholm DJ, Campbell JP. Predicting survival from out-of-hospital cardiac arrest. *Prehosp Disaster Med* 1998; **13**(2-4): 51-4.

84. Lindner TW, Soreide E, Nilsen OB, Torunn MW, Lossius HM. Good outcome in every fourth resuscitation attempt is achievable--an Utstein template report from the Stavanger region. *Resuscitation* 2011; **82**(12): 1508-13.

85. Lombardi G, Gallagher J, Gennis P. Outcome of out-of-hospital cardiac arrest in New York City. The Pre-Hospital Arrest Survival Evaluation (PHASE) Study. *Jama* 1994; **271**(9): 678-83.

86. Lui JC. Evaluation of the use of automatic external defibrillation in out-of-hospital cardiac arrest in Hong Kong. *Resuscitation* 1999; **41**(2): 113-9.

87. Lund I, Skulberg A. Cardiopulmonary resuscitation by lay people. *Lancet (London, England)* 1976; **2**(7988): 702-4.

88. Ma MH, Chiang WC, Ko PC, et al. Outcomes from out-of-hospital cardiac arrest in Metropolitan Taipei: does an advanced life support service make a difference? *Resuscitation* 2007; **74**(3): 461-9.

89. Mathiesen WT, Bjorshol CA, Kvaloy JT, Soreide E. Effects of modifiable prehospital factors on survival after out-of-hospital cardiac arrest in rural versus urban areas. *Crit Care* 2018; **22**(1): 99.

90. May S, Zhang L, Foley D, et al. Improvement in Non-Traumatic, Out-Of-Hospital Cardiac Arrest Survival in Detroit From 2014 to 2016. *J Am Heart Assoc* 2018; **7**(16): e009831.

91. Mitchell RG, Brady W, Guly UM, Pirrallo RG, Robertson CE. Comparison of two emergency response systems and their effect on survival from out of hospital cardiac arrest. *Resuscitation* 1997; **35**(3): 225-9.

92. Mitchell RG, Guly UM, Rainer TH, Robertson CE. Can the full range of paramedic skills improve survival from out of hospital cardiac arrests? *Journal of accident & emergency medicine* 1997; **14**(5): 274-7.

93. Morrison LJ, Verbeek PR, Vermeulen MJ, et al. Derivation and evaluation of a termination of resuscitation clinical prediction rule for advanced life support providers. *Resuscitation* 2007; **74**(2): 266-75.

94. Myerburg RJ, Fenster J, Velez M, et al. Impact of Community-Wide Police Car Deployment of Automated External Defibrillators on Survival From Out-of-Hospital Cardiac Arrest. *Circulation* 2002; **106**(9): 1058-64.

95. Nehme Z, Andrew E, Bernard S, Smith K. Impact of cardiopulmonary resuscitation duration on survival from paramedic witnessed out-of-hospital cardiac arrests: An observational study. *Resuscitation* 2016; **100**: 25-31.

96. Nehme Z, Bernard S, Cameron P, et al. Using a cardiac arrest registry to measure the quality of emergency medical service care: decade of findings from the Victorian Ambulance Cardiac Arrest Registry. *Circ Cardiovasc Qual Outcomes* 2015; **8**(1): 56-66.

97. Nichol G, Thomas E, Callaway CW, et al. Regional variation in out-of-hospital cardiac arrest incidence and outcome. *Jama* 2008; **300**(12): 1423-31.

98. Nishiuchi T, Hiraide A, Hayashi Y, et al. Incidence and survival rate of bystander-witnessed out-of-hospital cardiac arrest with cardiac etiology in Osaka, Japan: a population-based study according to the Utstein style. *Resuscitation* 2003; **59**(3): 329-35.

99. Ogawa T, Akahane M, Koike S, Tanabe S, Mizoguchi T, Imamura T. Outcomes of chest compression only CPR versus conventional CPR conducted by lay people in patients with out of hospital cardiopulmonary arrest witnessed by bystanders: nationwide population based observational study. *BMJ* 2011; **342**: c7106.

100. Ohshige K, Shimazaki S, Hirasawa H, et al. Evaluation of out-of-hospital cardiopulmonary resuscitation with resuscitative drugs: a prospective comparative study in Japan. *Resuscitation* 2005; **66**(1): 53-61.

101. Olasveengen TM, Lund-Kordahl I, Steen PA, Sunde K. Out-of hospital advanced life support with or without a physician: effects on quality of CPR and outcome. *Resuscitation* 2009; **80**(11): 1248-52.

102. Olasveengen TM, Wik L, Steen PA. Standard basic life support vs. continuous chest compressions only in out-of-hospital cardiac arrest. *Acta Anaesthesiol Scand* 2008; **52**(7): 914-9.

103. Ong ME, Ng FS, Anushia P, et al. Comparison of chest compression only and standard cardiopulmonary resuscitation for out-of-hospital cardiac arrest in Singapore. *Resuscitation* 2008; **78**(2): 119-26.

104. Ong ME, Shin SD, De Souza NN, et al. Outcomes for out-of-hospital cardiac arrests across 7 countries in Asia: The Pan Asian Resuscitation Outcomes Study (PAROS). *Resuscitation* 2015; **96**: 100-8.

105. Pepe PE, Levine RL, Fromm RE, Jr., Curka PA, Clark PS, Zachariah BS. Cardiac arrest presenting with rhythms other than ventricular fibrillation: contribution of resuscitative efforts toward total survivorship. *Critical care medicine* 1993; **21**(12): 1838-43.

106. Pleskot M, Babu A, Kajzr J, et al. Characteristics and short-term survival of individuals with out-of-hospital cardiac arrests in the East Bohemian region. *Resuscitation* 2006; **68**(2): 209-20.

107. Rainer TH, Gordon MW, Robertson CE, Cusack S. Evaluation of outcome following cardiac arrest in patients presenting to two Scottish emergency departments. *Resuscitation* 1995; **29**(1): 33-9.

108. Rajan S, Wissenberg M, Folke F, et al. Association of Bystander Cardiopulmonary Resuscitation and Survival According to Ambulance Response Times After Out-of-Hospital Cardiac Arrest. *Circulation* 2016; **134**(25): 2095-104.

109. Rea TD, Eisenberg MS, Culley LL, Becker L. Dispatcher-assisted cardiopulmonary resuscitation and survival in cardiac arrest. *Circulation* 2001; **104**(21): 2513-6.

110. Rea TD, Fahrenbruch C, Culley L, et al. CPR with chest compression alone or with rescue breathing. *N Engl J Med* 2010; **363**(5): 423-33.

111. Richless LK, Schrading WA, Polana J, Hess DR, Ogden CS. Early defibrillation program: problems encountered in a rural/suburban EMS system. *The Journal of emergency medicine* 1993; **11**(2): 127-34.

112. Ritter G, Wolfe RA, Goldstein S, et al. The effect of bystander CPR on survival of out-of-hospital cardiac arrest victims. *Am Heart J* 1985; **110**(5): 932-7.

113. Ro YS, Shin SD, Kitamura T, et al. Temporal trends in out-of-hospital cardiac arrest survival outcomes between two metropolitan communities: Seoul-Osaka resuscitation study. *BMJ Open* 2015; **5**(6): e007626.

114. Rudner R, Jalowiecki P, Karpel E, Dziurdzik P, Alberski B, Kawecki P. Survival after out-of-hospital cardiac arrests in Katowice (Poland): outcome report according to the "Utstein style". *Resuscitation* 2004; **61**(3): 315-25.

115. Schneider T, Mauer D, Diehl P, Eberle B, Dick W. Quality of on-site performance in prehospital advanced cardiac life support (ACLS). *Resuscitation* 1994; **27**(3): 207-13.

116. Shao F, Li CS, Liang LR, Li D, Ma SK. Outcome of out-of-hospital cardiac arrests in Beijing, China. *Resuscitation* 2014; **85**(11): 1411-7.

117. Smith JP, Bodai BI. Guidelines for discontinuing cardiopulmonary resuscitation in the emergency department after prehospital, nonparamedic-directed cardiac arrest. *The Western journal of medicine* 1985; **143**(3): 402-5.

118. Sondergaard KB, Wissenberg M, Gerds TA, et al. Bystander cardiopulmonary resuscitation and long-term outcomes in out-of-hospital cardiac arrest according to location of arrest. *Eur Heart J* 2019; **40**(3): 309-18.

119. Soo LH, Gray D, Young T, Huff N, Skene A, Hampton JR. Resuscitation from out-of-hospital cardiac arrest: is survival dependent on who is available at the scene? *Heart* 1999; **81**(1): 47-52.

120. Stapczynski JS, Svenson JE, Stone CK. Population density, automated external defibrillator use, and survival in rural cardiac arrest. *Acad Emerg Med* 1997; **4**(6): 552-8.

121. Steinmetz J, Barnung S, Nielsen SL, Risom M, Rasmussen LS. Improved survival after an out-of-hospital cardiac arrest using new guidelines. *Acta Anaesthesiol Scand* 2008; **52**(7): 908-13.

122. Stiell IG, Wells GA, DeMaio VJ, et al. Modifiable factors associated with improved cardiac arrest survival in a multicenter basic life support/defibrillation system: OPALS Study Phase I results. Ontario Prehospital Advanced Life Support. *Ann Emerg Med* 1999; **33**(1): 44-50.

123. Sunde K, Eftestol T, Askenberg C, Steen PA. Quality assessment of defribrillation and advanced life support using data from the medical control module of the defibrillator. *Resuscitation* 1999; **41**(3): 237-47.

124. Svensson L, Bohm K, Castren M, et al. Compression-only CPR or standard CPR in out-of-hospital cardiac arrest. *N Engl J Med* 2010; **363**(5): 434-42.

125. Swor RA, Jackson RE, Cynar M, et al. Bystander CPR, ventricular fibrillation, and survival in witnessed, unmonitored out-of-hospital cardiac arrest. *Ann Emerg Med* 1995; **25**(6): 780-4.

126. Swor RA, Jackson RE, Tintinalli JE, Pirrallo RG. Does advanced age matter in outcomes after out-of-hospital cardiac arrest in community-dwelling adults? *Acad Emerg Med* 2000; **7**(7): 762-8.

127. Tadel S, Horvat M, Noc M. Treatment of out-of-hospital cardiac arrest in Ljubljana: outcome report according to the 'Utstein' style. *Resuscitation* 1998; **38**(3): 169-76.

128. Tanaka H, Ong MEH, Siddiqui FJ, et al. Modifiable Factors Associated With Survival After Out-of-Hospital Cardiac Arrest in the Pan-Asian Resuscitation Outcomes Study. *Ann Emerg Med* 2018; **71**(5): 608-17 e15.

129. Tresch DD, Neahring JM, Duthie EH, Mark DH, Kartes SK, Aufderheide TP. Outcomes of cardiopulmonary resuscitation in nursing homes: can we predict who will benefit? *Am J Med* 1993; **95**(2): 123-30.

130. Valenzuela TD, Roe DJ, Cretin S, Spaite DW, Larsen MP. Estimating effectiveness of cardiac arrest interventions: a logistic regression survival model. *Circulation* 1997; **96**(10): 3308-13.

131. van der Hoeven JG, Waanders H, Compier EA, van der Weyden PK, Meinders AE. Prolonged resuscitation efforts for cardiac arrest patients who cannot be resuscitated at the scene: who is likely to benefit? *Ann Emerg Med* 1993; **22**(11): 1659-63.

132. Van Hoeyweghen RJ, Bossaert LL, Mullie A, et al. Quality and efficiency of bystander CPR. Belgian Cerebral Resuscitation Study Group. *Resuscitation* 1993; **26**(1): 47-52.

133. Waalewijn RA, de Vos R, Koster RW. Out-of-hospital cardiac arrests in Amsterdam and its surrounding areas: results from the Amsterdam resuscitation study (ARREST) in 'Utstein' style. *Resuscitation* 1998; **38**(3): 157-67.

134. Waalewijn RA, Tijssen JG, Koster RW. Bystander initiated actions in out-of-hospital cardiopulmonary resuscitation: results from the Amsterdam Resuscitation Study (ARRESUST). *Resuscitation* 2001; **50**(3): 273-9.

135. Westfal RE, Reissman S, Doering G. Out-of-hospital cardiac arrests: an 8-year New York City experience. *Am J Emerg Med* 1996; **14**(4): 364-8.

136. Weston CF, Jones SD, Wilson RJ. Outcome of out-of-hospital cardiorespiratory arrest in south Glamorgan. *Resuscitation* 1997; **34**(3): 227-33.

137. White RD, Bunch TJ, Hankins DG. Evolution of a community-wide early defibrillation programme experience over 13 years using police/fire personnel and paramedics as responders. *Resuscitation* 2005; **65**(3): 279-83.

138. Wilson BH, Severance HW, Jr., Raney MP, et al. Out-of-hospital management of cardiac arrest by basic emergency medical technicians. *Am J Cardiol* 1984; **53**(1): 68-70.

139. Wissenberg M, Lippert FK, Folke F, et al. Association of national initiatives to improve cardiac arrest management with rates of bystander intervention and patient survival after out-of-hospital cardiac arrest. *JAMA* 2013; **310**(13): 1377-84.

140. Yasunaga H, Horiguchi H, Tanabe S, et al. Collaborative effects of bystander-initiated cardiopulmonary resuscitation and prehospital advanced cardiac life support by physicians on survival of out-of-hospital cardiac arrest: a nationwide population-based observational study. *Crit Care* 2010; **14**(6): R199.

141. Yen ZS, Chen YT, Ko PC, et al. Cost-effectiveness of different advanced life support providers for victims of out-of-hospital cardiac arrests. *Journal of the Formosan Medical Association = Taiwan yi zhi* 2006; **105**(12): 1001-7.
